# Mortality burden of cardiovascular disease attributable to ambient PM_2.5_ exposure in Portugal, 2011 to 2021

**DOI:** 10.1186/s12889-024-18572-0

**Published:** 2024-04-27

**Authors:** Mariana O. Corda, Periklis Charalampous, Juanita A. Haagsma, Ricardo Assunção, Carla Martins

**Affiliations:** 1 NOVA National School of Public Health, Public Health Research Centre, NOVA University Lisbon, Lisbon, Portugal; 2 Department of Public Health, Erasmus MC, University Medical Center Rotterdam, Rotterdam, The Netherlands; 3 Institute of Health and Society (IRSS), Université Catholique de Louvain, Brussels, Belgium; 4 Egas Moniz Center for Interdisciplinary Research (CiiEM), Egas Moniz School of Health & Science, Caparica, Almada, Portugal; 5 CESAM, Center for Environmental and Marine Studies, University of Aveiro, Campus Universitário de Santiago, Aveiro, Portugal; 6 NOVA National School of Public Health, Public Health Research Centre, Comprehensive Health Research Center, CHRC, NOVA University Lisbon, Lisbon, Portugal

**Keywords:** Air pollution, Fine particles, Long-term exposure, Cardiovascular disease, Years of life lost, Burden of disease, Public health

## Abstract

**Background:**

Exposure to high levels of environmental air pollution causes several health outcomes and has been associated with increased mortality, premature mortality, and morbidity. Ambient exposure to PM_2.5_ is currently considered the leading environmental risk factor globally. A causal relationship between exposure to PM_2.5_ and the contribution of this exposure to cardiovascular morbidity and mortality was already demonstrated by the American Heart Association.

**Methods:**

To estimate the burden of mortality attributable to environmental risk factors, a comparative risk assessment was performed, considering a “top-down” approach. This approach uses an existing estimate of mortality of the disease endpoint by all causes as a starting point. A population attributable fraction was calculated for the exposure to PM_2.5_the overall burden of IHD and stroke was multiplied by the PAF to determine the burden attributable to this risk factor. The avoidable burden was calculated using the potential impact fraction (PIF) and considering the WHO-AQG 2021 as an alternative scenario.

**Results:**

Between 2011 and 2021, the ambient exposure to PM_2.5_ resulted in a total of 288,862.7 IHD YLL and a total of 420,432.3 stroke YLL in Portugal. This study found a decreasing trend in the mortality burden attributable to PM2.5 exposure, for both males and females and different age-groups. For different regions of Portugal, the same trend was observed in the last years. The mortality burden attributable to long-term exposure to PM_2.5_ was mainly concentrated in Lisbon Metropolitan Area, North and Centre. Changes in the exposure limits to the WHO recommended value of exposure (WHO-AQG 2021) have a reduction in the mortality burden due to IHD and stroke attributable to PM_2.5_ exposure, in Portugal.

**Conclusion:**

Between 2011 and 2021, approximately 22% and 23% of IHD and stroke deaths were attributable to PM_2.5_ exposure. Nevertheless, the mortality burden attributable to cardiovascular diseases has been decreasing in last years in Portugal. Our findings provide evidence of the impact of air pollution on human health, which are crucial for decision-making, at the national and regional level.

**Supplementary Information:**

The online version contains supplementary material available at 10.1186/s12889-024-18572-0.

## Background

Exposure to high levels of environmental air pollution has been associated with increased mortality, premature mortality, and morbidity [1]. In fact, air pollution is one of the major risk factors for human health and ranks just below hypertension, tobacco use, and dietary risks, and is the leading cause of death due to environmental risk factor worldwide, affecting everyone in low-, middle-, and high-income countries [2, 3]. The World Health Organization (WHO) reported that the effects of ambient and household air pollution combined are responsible for 7 million premature deaths annually [4, 5]. Ambient air pollutants, like ozone (O_3_), nitrogen dioxide (NO_2_), or particulate matter (PM) have been associated with several health impacts [6]. Heart disease and stroke are the most common cause of premature deaths attributable to air pollution, followed by lung disease and lung cancer [7].

Ambient particulate matter (PM) is a major component of air pollution, comprising a varied blend of particles with different dimensions and chemical compositions. PM is the sixth most significant risk factor for global mortality and was responsible for more than two million of cardiovascular deaths in 2019 [8]. Particulate matter (PM) with a diameter of 2.5 μm or less, usually named PM_2.5_, is one of the air pollutants more harmful to human health [6]. These particles are inhaled, penetrate deep into the respiratory tract, and cause systematic inflammation and oxidative stress followed by direct translocation into the systemic circulation and perturbation of the autonomic nervous system [9, 10]. These processes contribute to the progression of atherosclerosis [11], the pathophysiological process behind IHD and strokes.

Long-term exposure to this pollutant is associated with several health outcomes, including stroke, ischemic heart disease (IHD), chronic obstructive pulmonary disease, lung cancer, and acute lower respiratory infection [12–14]. Indeed, a causal relationship between exposure to PM_2.5_ and the contribution of this exposure to cardiovascular morbidity and mortality was demonstrated by the American Heart Association (AHA) [4, 15]. In 2021, exposure to fine PM (PM_2.5_) concentrations above limits defined by WHO guidelines was responsible for 253 000 deaths in the Europe (EU) and 2 100 deaths in Portugal [16]. However, the health impact in some countries, such as Portugal, is not well characterized. While previous studies have investigated the long-term exposure to PM_2.5_ and its association with mortality from cardiovascular disorders in Portugal [17, 18]. there remains a notable gap regarding a comprehensive analysis that includes the calculation of years of life lost (YLL). The main objective of this study was to estimate the environmental burden of disease (EBD) of IHD and stroke attributable to exposure to PM_2.5_ in mainland Portugal and respective regions, in terms of years of life lost due to premature mortality between 2011 and 2021. The evaluation of YLL with different scenarios of exposure will provide scientific evidence for future policy actions aiming to protect human health.

## Methods

### Study design

A descriptive, longitudinal, and retrospective study was performed to estimate the environmental mortality burden in mainland Portugal. Located in southwest Europe, mainland Portugal has a population of 9.86 million people and an area of 88,889 square kilometres. According to the territorial division system, mainland Portugal is divided into five regions classified as Nomenclature of Territorial Units for Statistical Purposes II (NUTS II), North, Centre, Lisbon Metropolitan Area, Alentejo, and Algarve. For this study, the Portuguese adult population, older than 30 years and for both sexes, males, and females, was considered. The Autonomous regions of Madeira and Azores were excluded from the study area; in this way, when Portugal is mentioned, it refers to mainland Portugal.

### Data sources

Validated hourly concentrations of PM_2.5_ and PM_10_ from January 2011 to December 2021 in Portugal were obtained from the Portuguese Environment Agency (APA) website (https://qualar.apambiente.pt/) and the regional allocated PM_2.5_ concentrations were acquired as previously reported by Lima [19]**.** Briefly, the annual average of PM_2.5_ and PM_10_ was estimated based on the concentrations of each station, using the previous 365 days, and using only the station with a minimum reporting percentage of 75% to ensure the uniformity of the results. In the absence of PM_2.5_ data, the PM_10_ data were used to estimate the PM_2.5_ concentration using the ratio PM_2.5_/PM_10_ = 0.65 [20]. The monitoring data was organized by different areas of Portugal at the NUTS II level.

The demographic data were obtained from Statistics Portugal, from the annual report (2011—2021) [21].

The mortality data were extracted from Statistics Portugal [22], grouped by cause of death, using the European short-list for causes of death. All deaths with International Classification of Diseases, ninth and tenth Revision, Clinical Modification (ICD-9-CM and ICD-10-CM) codes 410 to 414.9 and I20 to I25.9 for IHD and 430 to 438.9 and I60 to I69.8 for stroke were considered. The cause-of-death data were stratified by sex (i.e., males and females), five-year age groups (i.e., 30–34 y; 35–39 y; 40–44 y; 45–49 y; 50–54 y; 55–59 y; 60–64 y; 65–69 y; 70–74 y; 75–79 y; 80–84 y; and 85 + y), and for five different regions of Portugal (i.e., North, Centre, Lisbon Metropolitan Area, Alentejo, and Algarve), and years (i.e., from 2011 to 2021).

### Mortality-related EBD and scenarios of exposure

To estimate the burden of mortality attributable to environmental risk factors, a comparative risk assessment (CRA) approach was performed, considering a “top-down” approach [23]. This approach uses an existing estimate of mortality of the disease endpoint by all causes as a starting point. A population attributable fraction (PAF) was calculated for the exposure to PM_2.5_ and the overall burden of IHD and stroke was multiplied by the PAF to determine the burden attributable to this risk factor [24, 25].

**Fig. 1:**
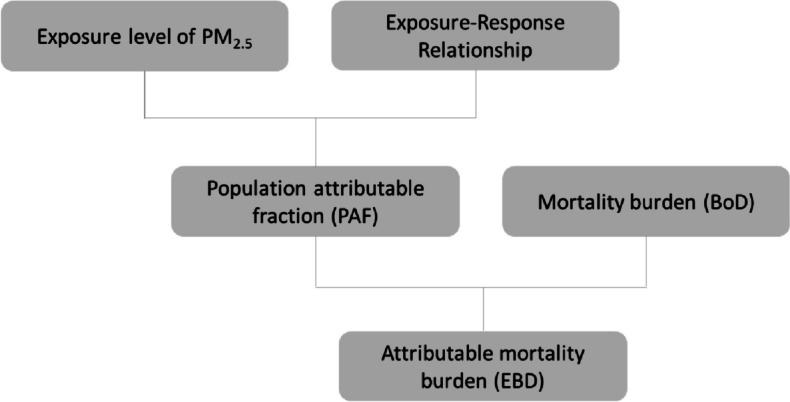
Flow chart of the processes leading to an estimate of the burden of cardiovascular diseases attributable to PM_2.5_ exposure (source: adapted from Plass et al. [24])

In this study, we estimated the burden of IHD, and stroke mortality attributed to exposure to PM_2.5_ by sequentially estimating environmental exposure assessment; total burden of cardiovascular mortality (BoD); population attributable fraction (PAF); and the attributable burden of mortality (EBD) (Fig. 1).

For the years 2019 and 2021, the burden of mortality, estimated in YLL, was calculated under current PM_2.5_ levels (scenario 1, S1) and for four different scenarios of PM_2.5_ levels: the higher level of PM_2.5_ in 2021 (scenario 2, S2), WHO Air Quality Guidelines (WHO-AQG) recommended in 2005 [26] (scenario 3, S3), WHO-AQG recommended in 2021 [27] (scenario 4, S4) and probabilistic analysis, where estimates reflected the air monitoring data distribution (scenario 5, S5) (Table 1).

**Table 1:** Description of the different considered scenarios, respective PM_2.5_ concentrations (*µg/m*^*3*^*),* and years for which the analysis was carried out

*Scenario*	*Description*	*PM*_*2.5*_* concentration* *(µg/m*^*3*^*)*	*Time frame*
1	Annual mean concentrations	-	2011 – 2021
2	Higher level of PM_2.5_ in 2021	147.5	2019 and 2021
3	2005 WHO Air Quality Guidelines	10	2019 and 2021
4	2021 WHO Air Quality Guidelines	5	2019 and 2021
5	Probabilistic analysis (@Risk)	[PM_2.5_] probabilistic distribution	2019 and 2021

*[PM2.5]* PM_2.5_ concentration

### Total burden of IHD and stroke mortality

To estimate the YLL due to IHD and stroke, the number of deaths in each age group was multiplied by the remaining life expectancy at the age of death [28].

1 $$YLL\ (BoD) = Number\ of\ deaths \times remaining\ life\ expectancy\ (RLE)$$

The remaining life expectancy (RLE) was provided by the GBD 2019 study [29]. Life expectancy at the age at which death occurs is equal for males and females. The total of YLLs for each gender was obtained by summing the YLLs of all age groups. YLL rate was calculated for 100,000 inhabitants (YLL/100k).

### Population attributable fraction and attributable burden of mortality

The population attributable fraction (PAF) is used as a metric to assess the contribution of a risk factor to a disease or a death [24]. The PAF allows us to estimate the total mortality burden attributable to PM_2.5_ exposure in the population under study when the exposure was assumed as theoretical minimal risk exposure level, counterfactual concentration (C_0_), of 0 µg/m^3^ (considering the absence of evidence of a threshold below which air pollution does not impact health [16, 30]). The relative risk (RR_c_) in a population whose exposure is estimated by an average concentration C can be described as a log-linear function relating concentrations and mortality according to the Eq. 2:

2 $${RR}_{c}= {e}^{[\beta \times \left(C-{C}_{0}\right)]}$$

where, RRc is the RR of the specific exposure concentration of PM_2.5_ in each scenario, C is the concentration level to which the population is exposed, C_0_ is the counterfactual concentration, and β, a log-linear slop, and is based on the concentration response-function (CRF). The CRF was used to quantify the association between the outcome and the risk, i.e. measure the effect per unit increase in exposure (dose), quantitatively. The CRFs for both causes of death are derived from the literature that estimated the effect measure (RR_literature_) [31]. For IHD mortality the RR_literature_ = 1.23 (CI 95% 1.15–1.31) per 10 µg/m^3^ increase (dose) in long-term PM_2.5_ and for stroke mortality the RR_literature_ = 1.24 (CI 95% 1.13–1.36) per 10 µg/m^3^ increase (dose) in long-term PM_2.5._ The β can be estimated as follows:

3 $$\beta =\frac{{\text{ln}}\ {RR}_{literature}}{Dose}$$

The PAF was estimated for Portugal and for different ages throughout the years under study and is not sex or age specific. To calculate the PAF the following formula (Eq. 4) was used:

4 $$PAF= \frac{{RR}_{c}-1}{{RR}_{c}}$$

Finally, to estimate the mortality related disease burden attributable to a specific risk factor (YLL (EBD)), ambient PM_2.5_, the YLL (BoD) estimates was multiplied by PAF (Eq. 5):

5 $$YLL \left(EBD\right)=YLL\left(BoD\right)\times PAF$$

The calculations for scenario 5 (probabilistic approach) employed the same model, with the inclusion of input data variability. This was accomplished by representing selected variables with probabilistic distributions, chosen based on the raw data. Regarding PAF, the distributions presenting a better-adjusted fit were the triangular distribution for the RR_literature_ to estimate the value of β, and the exponential distribution to estimate the probable concentration of PM_2.5_. Further details about this analysis are provided in supplementary material (Table S4).

### Avoidable burden of cardiovascular disease

The potential impact fraction (PIF) is a measure of the proportional change in disease burden after a change in exposure to a related risk factor [32]— in this case, changes in exposure to PM_2.5_ from the current (reference scenario) to alternative scenario — and was calculated for the alternative scenario of WHO-AQG 2021 (S4). This analysis was done for 2019 and 2021, and the calculations were performed following the formula (Eq. 6).

6 $$PIF= \frac{{\sum }_{i=1}^{1}{RR}_{ref}- {\sum }_{i=1}^{1}{RR}_{alt}}{{\sum }_{i=1}^{1}{RR}_{ref}}$$

where RR_ref_ is the RR of the reference scenario and RR_alt_ is the RR of the alternative scenario (*n* = 1).

The calculations for the scenario 5 (probabilistic approach) considered a uniform distribution since it was considered a fixed value for PM_2.5_. Further details about this analysis are provided in supplementary material (Table S4).

### Statistical analysis

All estimations were performed using statistical computing Microsoft Excel (version 2307 Build 16. 0. 16,626. 20,170). The scenarios S1, S2, S3 and S4 considered point estimates applying a deterministic approach, with data sources and calculations above mentioned. For the scenario 5, a probabilistic approach was considered for all calculations to include the inherent variability of PM_2.5_ concentration parameters, using the @RISK (Palisade, version 8.5.1) software. The Akaike Information Criterion (AIC) was used to select the better-fit distribution model. The results were obtained through a Monte Carlo simulation with 100,000 iterations. For the probabilistic approach, values for mean, median (p50), percentile 2.5 (P2.5) and percentile 97.5 (P97.5) (95% of observations), and percentile 75 (P75) were extracted.

## Results

### Ambient PM_2.5_ exposure assessment and population attributable fraction

The exposure of the Portuguese population to PM_2.5_ was estimated considering the reported PM_2.5_ concentration levels. The annual average concentration of PM_2.5_ in Portugal and its different regions between 2011 and 2021 has been decreasing. However, the level of pollution by PM_2.5_ still higher than the recommended by WHO guidelines (2021) [27] (Table S1). The highest levels of PM_2.5_ were observed for North, Centre, Lisbon Metropolitan Area, and Algarve in 2011, and for mainland Portugal and for Alentejo was registered the highest level in 2013. Between 2011 and 2021, in Portugal was verified a decrease of -41.8% in PM_2.5_ levels. Algarve was the region with the highest decrease in levels of this pollutant (-60,9%), followed by Lisbon Metropolitan Area (-41.8%), Alentejo (-39.7%), Centre (-35.5%) and North (-30.4%). Further details are presented in Table S1. Table 2 presents the information regarding the population attributable fraction (PAF) linked to PM_2.5_ exposure.

**Table 2:** Percentage of population attributable fraction (PAF), for each cause of death, for Portugal and its different regions, 2011–2021

	**PAF (Ischemic Heart Disease) (%)**						**PAF (Stroke) (%)**					
	**Portugal**	**North**	**Centre**	**LMA**^**a**^	**Alentejo**	**Algarve**	**Portugal**	**North**	**Centre**	**LMA**^**a**^	**Alentejo**	**Algarve**
**2011**	28.9	28.7	27.8	31.5	21.8	26.0	29.8	29.6	28.7	32.5	22.6	26.9
**2012**	24.5	23.8	25.9	26.7	19.0	18.6	25.3	24.6	26.8	27.6	19.6	19.3
**2013**	23.9	22.8	24.6	26.8	22.7	19.9	24.8	23.6	25.5	27.7	23.5	20.6
**2014**	21.6	15.6	22.2	22.9	21.9	19.0	22.4	16.1	23.0	23.7	22.7	19.6
**2015**	23.5	21.3	22.0	26.1	20.1	23.7	24.3	22.0	22.8	26.9	20.8	24.5
**2016**	21.4	21.5	18.6	24.1	16.7	22.2	22.1	22.3	19.3	24.9	17.3	23.0
**2017**	23.2	20.9	20.4	25.8	20.9	24.2	24.0	21.7	21.1	26.6	21.6	25.0
**2018**	21.4	20.1	19.1	24.2	13.3	24.2	22.1	20.8	19.8	25.1	13.8	25.0
**2019**	19.5	17.3	18.6	21.8	11.5	23.7	20.2	18.0	19.3	22.5	11.9	24.5
**2020**	17.8	21.5	16.0	19.8	12.1	16.3	18.4	22.6	16.6	20.5	12.5	16.9
**2021**	18.0	21.0	18.9	19.7	13.8	11.1	18.6	21.7	19.6	20.4	14.3	11.5

^a^*LMA* Lisbon Metropolitan Area

As expected, the percentage of PAF had decreased for Portugal and for all regions, between 2011 and 2021. The highest decrease in PAF values for IHD and stroke was observed in Lisbon Metropolitan Area and the lowest decrease in PAF values was observed for Alentejo.

### Environmental burden of disease attributable to PM_2.5_ exposure, 2011–2021

Between 2011 and 2021, in Portugal, a total of 16,276 and 28,537 of deaths due to IHD and stroke attributable to PM_2.5_ exposure were estimated, respectively, which represent approximately 22% and 23% of the total number of deaths due to IHD and stroke, respectively. These deaths attributable to PM_2.5_ exposure represent a total of 288,862.7 IHD YLL and a total of 420,432.3 stroke YLL, for Portugal and for both sexes (Table 3).

**Table 3:** Total of years of life lost (YLL) and YLL rates per 100,000 inhabitants (100 K) for each cause of death by different variables (sex, region, and age-group), 2011–2021

	**Ischemic Heart Disease**		**Stroke**	
	YLL	YLL rates (100 K)	YLL	YLL rates (100 K)
**Portugal**	288,862.7	4,075.1	420,432.3	5,931.1
**Sex**				
Males	183,973.0	5,607.6	202,233.0	6,164.2
Females	104,889.7	2,754.6	218,199.3	5,730.4
**Region**				
North	78,507.2	3,036.2	134,796.9	5,213.1
Centre	53,944.2	3,262.0	107,442.5	6,497.1
LMA^a^	114,815.0	5,763.8	121,960.8	6,135.0
Alentejo	23,768.0	4,564.3	32,309.6	6,204.6
Algarve	15,910.4	4,729.7	16,119.1	4,791.8
**Age-group**				
30–34	1,448.2	270.1	1,175.9	219.3
35–39	3,413.1	567.4	2,493.7	414.5
40–44	7,437.9	1,054.4	4,799.0	680.3
45–49	12,210.9	1,592.1	7,823.2	1,020.0
50–54	17,781.1	2,481.2	11,536.9	1,609.9
55–59	21,906.6	3,090.9	15,783.4	2,226.9
60–64	26,483.6	3,880.7	21,489.4	3,148.9
65–69	29,134.9	4,623.8	30,181.6	4,789.9
70–74	32,441.2	5,587.0	43,416.1	7,477.1
75–79	37,373.5	8,090.6	64,553.9	13,974.6
80–84	39,286.4	11,331.3	80,790.2	23,302.2
85 +	59,945.3	17,072.5	136,389.0	38,843.9

^a^*LMA* Lisbon Metropolitan Area

Between 2011 and 2021, in Portugal, males presented the highest crude number of IHD YLL (183,973.0) attributable to exposure to PM_2.5_, when compared to females (104,889.7) (Table 3). For stroke, females had the highest crude number of YLL attributable to exposure to PM_2.5_ (218,199.3), compared to males (202,233.0) (Table 3).

Across all the years, the total crude number of YLL attributable-PM_2.5_ by different age-groups were presented in Table 3. Overall, the number of YLLs due to IHD and stroke, in Portugal, increased with age. The number of YLL for IHD and stroke reached a peak at the 85 + age-group. The IHD presented a higher number of YLLs for the youth age-group (30–64 age-group) compared to stroke, however, for the older age-groups (65–85 +) stroke presented a higher number of YLLs (Table 3).

Comparing the different regions of Portugal, for the years 2011–2021, the Lisbon Metropolitan Area had the highest number of IHD YLL (114,815.0) and the Algarve had the lowest IHD YLL (15,910.4). However, when IHD YLL rates was considered, North was the regions with lowest mortality burden (Table 3). For stroke, the North was the region of Portugal with a higher number of YLL (78,507.2) and Algarve was the region with a lower number of YLL (16,119.1) (Table 3). But, when stroke YLL rate was considered, Centre was the region with higher mortality burden (6,497.1) (Table 3).

In Portugal, the highest IHD YLL and YLL rates were observed in 2011. For different regions, were registered higher values in IHD YLL and YLL rates for North in 2017, Centre, Lisbon Metropolitan Area and Alentejo in 2011 and Algarve in 2018 (Table S2). For stroke, the highest YLL and YLL rates were observed in 2011, in Portugal and all regions (Table S3).

### Trends of mortality-related EBD, over years 2011–2021

**Fig. 2:**
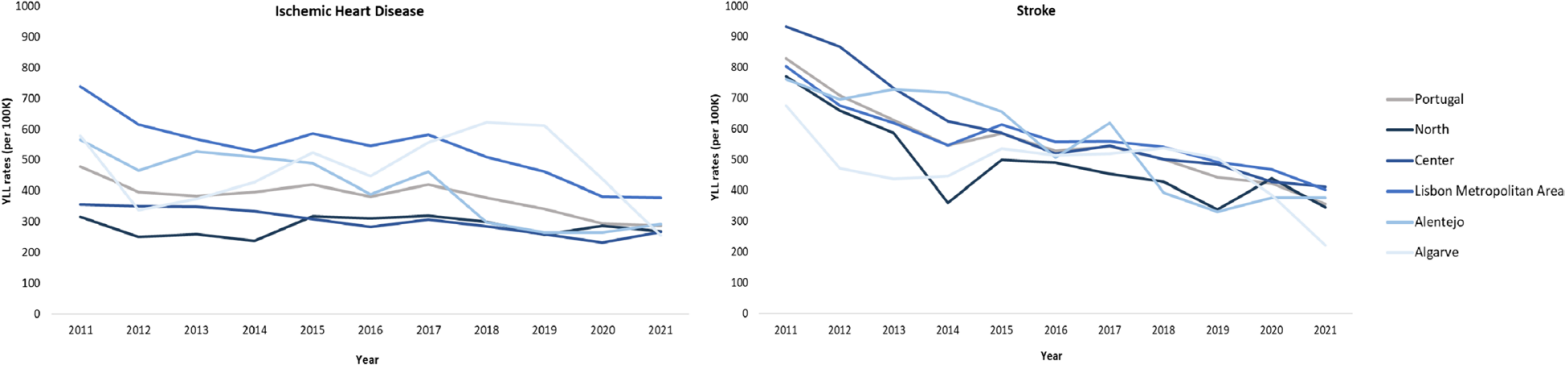
IHD and Stroke YLL rates per 100,000 inhabitants (100K) for different regions of Portugal, 2011–2021

Between 2011 and 2021 for both causes of death, a decrease was observed for the YLL rates per 100,000 inhabitants (100K), for Portugal and its different regions (Fig. 2). For Portugal, stroke was the cause of death that had a higher decrease of YLL/100K (-57.0%), approximately 1.4 times as for IHD YLL/100k (-40.0%). Algarve had the highest decrease of IHD YLL/100K and stroke YLL/100K, –55.9% and -67.2%, respectively. The regions North and Lisbon Metropolitan Area had the lowest decrease of IHD YLL/100K and stroke YLL/100K, -15.1% and -49.9%, respectively.

**Fig. 3:**
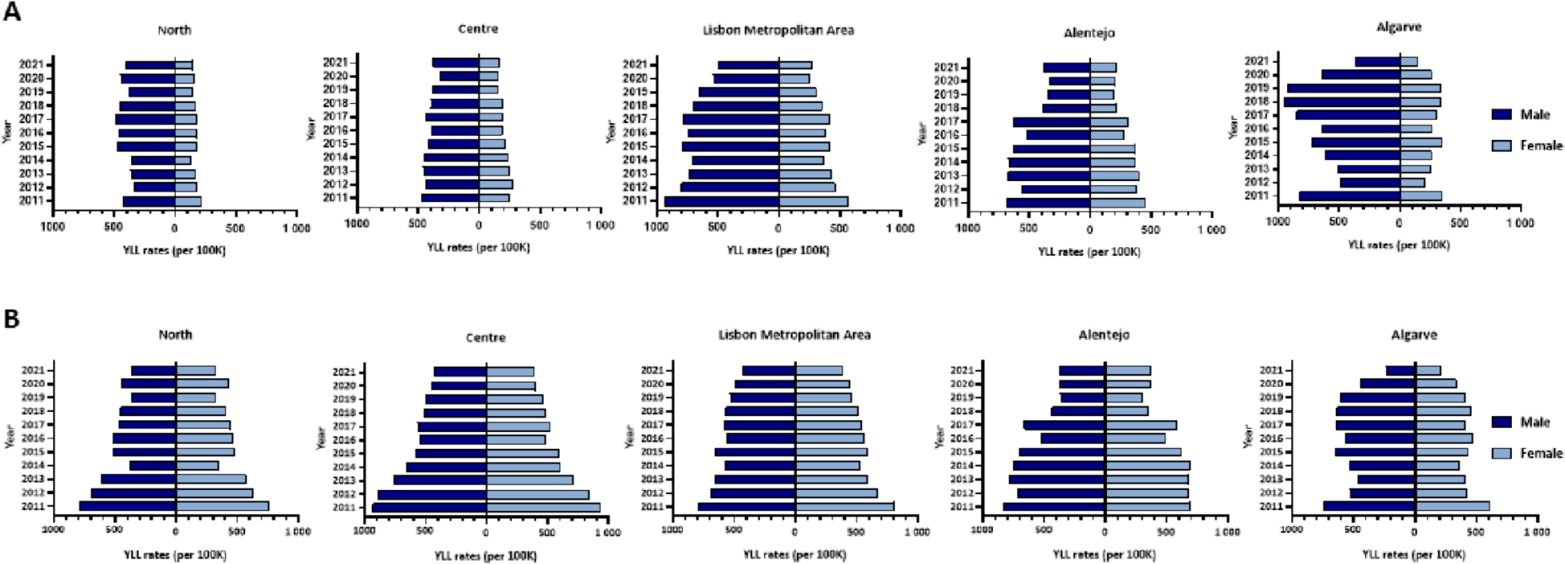
**A** IHD YLL rates (per 100 K) and (**B**) Stroke YLL rates (per 100 K) by regions of Portugal and sex, 2011–2021

Regarding region and sex, the IHD YLL rates decreased for males in Portugal (-35.1%) and in the different regions (-4.4% in North, -20.0% in Centre, -46.7% in Lisbon Metropolitan Area, -44.6% in Alentejo and -55.3% in Algarve). The IHD YLL rates decreased for females in Portugal (-47.3%) and for all its regions (-33.4% in North, -33.7% in Centre, -51.6% in Lisbon Metropolitan Area, -53.2% in Alentejo and -57.5% in Algarve). For both males and females, the highest decrease of IHD YLL/ 100 K was observed in Algarve (Fig. 3A).

The stroke YLL rates decreased for males in Portugal (-55.8%) and in the different regions, with Algarve being the region where the highest decrease was observed (-68.7%) compared with the four remaining regions of Portugal (-53.6% in North, -54.0% in Centre, -46.6% in Lisbon Metropolitan Area and -55.2% in Alentejo). For females, a decreasing trend was also observed for stroke YLL rates in Portugal (-58.1%) and in the different regions, with Algarve being the region with the highest decrease (–65.6%) compared with the other regions of Portugal (-56.5% in North, -57.7% in Centre, -52.6% in Lisbon Metropolitan Area and -45.4% in Alentejo) (Fig. 3B).

**Fig. 4:**
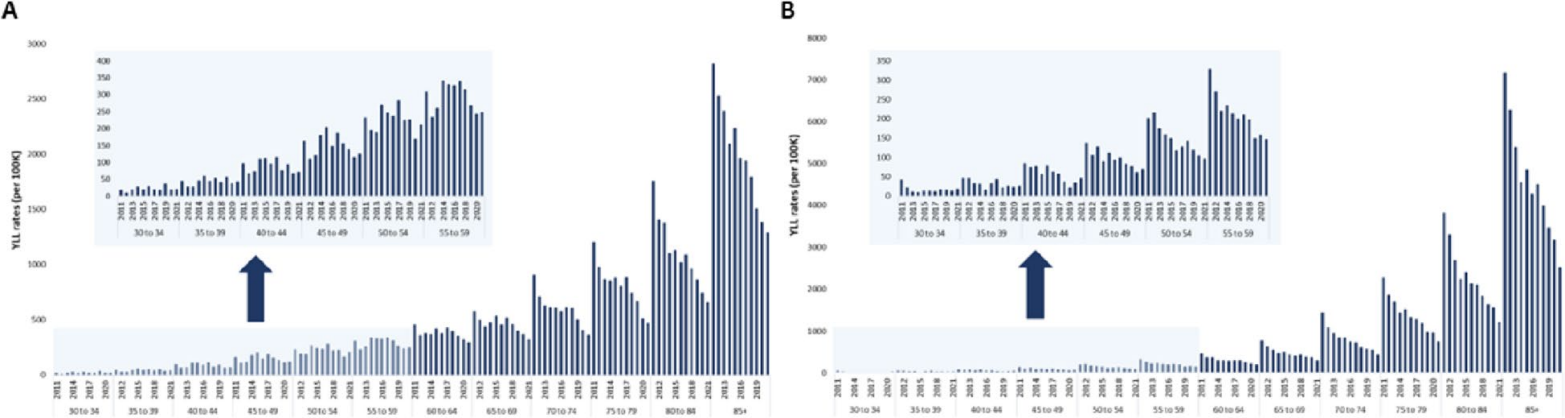
**A** IHD YLL rates (per 100 K) and (**B**) Stroke YLL rates (per 100 K) by age groups, 2011–2021, in Portugal

Figure 4 shows the YLL rates per 100 K inhabitants by age-group and causes of death, for both sexes in mainland Portugal, between 2011 and 2021.

Regarding age-groups, it was observed a decrease in the IHD YLL rate for all age-groups except for the age-group 30–34 for which an increase of + 11.7% in the YLL rate was observed (Fig. 4A). The highest decrease of IHD YLL rate was observed in age-group of 80-84y (-62.3%) compared to other age-groups (-4.0% at 35 to 39y, -25.5% at 40-44y, -23.5% at 45-49y, -9.1% at 50-54y, -19.7% at 55-59y, -34.6% at 60-64y, -43.9% at 65-69y, -59.8% at 70-74y, -61.2% at 75-79y and -54.1% at 85 + y). For stroke, it was observed a decrease in the YLL rates for all age-group (Fig. 4B). The highest decrease of stroke YLL/100 K was observed at age-group of 70-74y (-70.1%) compared to remaining age-groups (-56.3% at 30-34y, -43.0% at 35-39y, -45.0% at 40-44y, -49.2% at 45-49y, -51.6% at 50-54y, -55.2% at 55-59y, -57.4% at 60-64y, -60.5% at 65-69y, -67.1% at 75-79y, -68.3% at 80-84y, -64.8% at 85 +). For IHD, over time, for age-groups between 60 years and older than 85 years, a continuous decrease in the YLL rates was observed. However, for the other age-groups, the IHD YLL/100 K peak was observed in 2019 for 30-34y, in 2015 for 35-39y and 45-49y, in 2017 for 40-44y and 50-54y, and in 2014 for 55-59y. For stroke, a continuous decrease of YLL/100 K was observed for all age-groups with the highest YLL rates observed in 2011.

For all different regions of Portugal, the IHD YLL/100 K decreased for all age-groups from 60 years onwards. For the North and Lisbon Metropolitan Area the highest decrease was observed for age-group of 75-79y (-59.3% and -69.2%, respectively), for Centre was at 80-84y (-55.9%), for Alentejo was at 70-74y (-74.9%) and for Algarve was at 65-69y (-70.9%) (Figure S1). Lisbon Metropolitan Area, was the only region, were the continuous reduction of YLL rates for all age-groups were observed. The Centre was the region of Portugal with more fluctuations in IHD YLL rates. For stroke, in Lisbon Metropolitan Area, Alentejo and Algarve were observed an increase of the YLL rates (3.4% at 30-34y, 45.1% at 30-34y and 100% at 35-39y, respectively), and for North and Centre, only reductions of the YLL rates were observed (Figure S1). The highest decrease of stroke YLL/100 K was observed for age-group in North and Algarve at 30-34y (87.9% and 100%, respectively), in Centre and Lisbon Metropolitan Area at 80-84y (68.9% and 65.7%, respectively) and in Alentejo at 40-44y (86.7%).

### Health risk assessment for different scenarios of exposure

For 2019 and 2021, an analysis was carried out of the health impact of exposure to PM_2.5_ in different exposure scenarios (Table 1), which allowed us to compare the impact of exposure to annual levels of PM_2.5_ with the impact of exposure to high levels of pollution, the recommended exposure levels and with the probabilistic approach, in Portugal. Table 4 shows the PM_2.5_ exposure assessment considered for the different scenarios of exposure for 2019 and 2021.

**Table 4:** Exposure concentration of PM_2.5_ and population attributable fraction for different causes of death, for different scenarios (S1 to S5) by years, 2019 and 2021

			***S1***	***S2***	***S3***	***S4***	***S5***
***2019***	***[PM***_***2.5***_***] (µg/m***^***3***^***)***		10.5	147.5	10.0	5.0	10.5
	***PAF (%)***	***Stroke***	19.5	95.0	19.0	10.0	17.8
		***IHD***	20.2				18.5
***2021***	***[PM***_***2.5***_***] (µg/m***^***3***^***)***		9.6	147.5	10.0	5.0	9.6
	***PAF (%)***	***IHD***	18.0	95.0	19.0	10.0	16.6
		***Stroke***	18.6				17.2

*IHD* ischemic heart disease, *PAF* population attributable fraction, *S1* annual mean concentration, *S2* higher level of PM_2.5_ in 2021, *S3* 2005 WHO Air Quality Guidelines, *S4* 2021 WHO Air Quality Guidelines, *S5* Probabilistic analysis (@Risk)

**Fig. 5:**
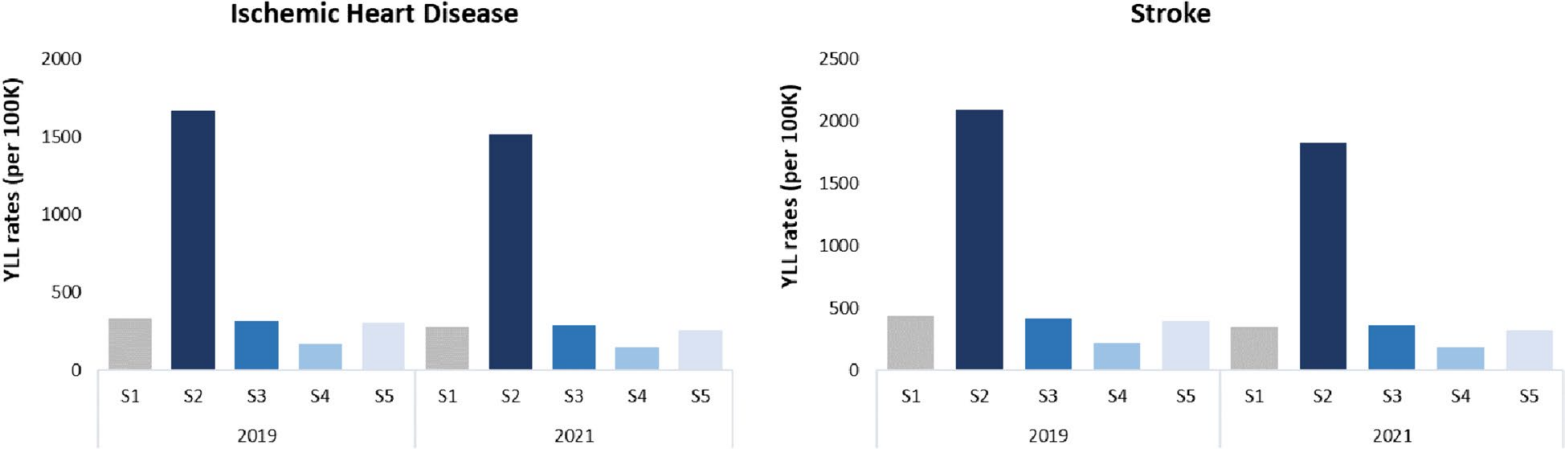
YLL rates per 100,000 inhabitants by cause of death, for each scenario, for Portugal and both sexes

Figure 5 shows the health impact of PM_2.5_ exposure for different scenarios assessed, for Portugal, in IHD and stroke YLL rates, for 2019 and 2021.

The exposure to higher levels as those registered in 2021 resulted in a higher number of YLL rates, for both causes of death, five times more compared with the values observed in scenario 1 for Portugal, in 2019 and 2021 (Fig. 5). Comparing the results obtained in S1 with the recommended exposure levels by WHO in 2021 (5 µg/m^3^) (S4), there is a reduction in IHD and stroke YLL rates of approximately half. However, when levels of exposure of 10 µg/m^3^ are considered (S3), there is no difference in the health impact if compared to the current scenario (S1) (Fig. 5). For the probabilistic analysis, S5, the exposure to the most probable value of PM_2.5_ mean concentration (Table S5) results in 313.0 YLL/100 K (P2.5 = 9.5; P97.5 = 980.8) and 264.6 YLL/100 K (P2.5 = 7.9; P97.5 = 839.2) in 2019 and 2021, respectively for IHD, and for stroke 405.0 YLL/100 K (P2.5 = 12.3; P97.5 = 1267.9) and 328.9 YLL/100 K (P2.5 = 7.9; P97.5 = 839.2), in 2019 and 2021, respectively. Additional results of the probabilistic approach can be found in the Supplementary Material (Table S6).

Comparing the health impact in the pre-pandemic period, 2019, and during the pandemic period in 2021, the YLL/100 K had decreased in 2021 for Portugal for both causes of death. Stroke was the cause of death for which the highest decrease in YLL rate (19.4%) was observed compared to IHD (15.9%).

### Avoidable environmental burden

An analysis of the avoidable burden was carried out for 2019 and 2021, allowing us to estimate the avoidable YLL rates if the WHO recommended 2021 PM_2.5_ levels (WHO-AQG 2021- 5µg/m^3^ (S4)) were adopted, in Portugal.

If the PM_2.5_ levels were within the annual limit of 5 µg/m^3^ as recommended by WHO (WHO-AQG 2021) this would have prevented 180.6 and 144.8 YLL/100K of IHD and 243.1 and 179.5 YLL/100K of stroke, in 2019 and 2021, respectively (Table 5). For estimated avoidable burden, the mortality burden of stroke was the one reducing the most compared to the burden of IHD. Considering a probabilistic approach, stroke was the cause of death with higher avoidable burden compared with IHD (Table 5). Additional results of the probabilistic approach can be found in the Supplementary Material (Table S7).

**Table 5:** Potential impact fraction and avoidable YLL/100 K for different causes of death and for alternative scenario 4 (WHO-AQG 2021) by year, 2019 and 2021 using a deterministic and a probabilistic approach

		**Deterministic**		**Probabilistic**	
		**IHD**	**Stroke**	**IHD**	**Stroke**
**2019**	***PIF (%)***	10.7	11.1	8.9	9.2
	***Avoidable YLL/100K***	180.6	243.1	150.1	201.7
**2021**	***PIF (%)***	9.06	9.4	7.5	7.8
	***Avoidable YLL/100K***	144.8	179.5	119.7	148.2

*PIF* potential impact fraction, *YLL* years of life lost, *IHD* ischemic heart disease

## Discussion

The ambient PM_2.5_ is the major environmental risk factor for human health with impact in cardiovascular mortality [3]. This study aimed to estimate the mortality burden of cardiovascular disease attributable to the exposure to PM_2.5_ in Portugal, between 2011 and 2021. We estimated a total of 288,862.7 IHD YLL and a total of 420,432.3 stroke YLL attributable to exposure to ambient PM_2.5_, across all years (2011–2021). These numbers represent around 22% and 23% of the total number of YLL due to IHD and stroke in Portugal, respectively.

PM_2.5_-attributable disease burden is higher in males than in females when IHD was considered as the cause of death. However, when stroke is considered as the cause of death, females had a higher number of YLL than males. While studies have investigated sex difference in the impact of PM_2.5_ exposure on cardiovascular disease [33, 34], evidence regarding mortality attributable to PM_2.5_ exposure remains inconclusive [34]. The variations observed in the mortality burden attributed to exposure between sexes can be explained by the differences in the overall mortality patterns for each cause of death. This is due to, the estimation of PAF considers the entire population rather than being sex-specific. The sex differences reported in our results is in accordance with the results of GBD 2019 study [8] when IHD is considered as cause of death, in contrast to our results for stroke. Difference in the methodology might explain this difference.

For both causes of death, the disease burden attributable to PM_2.5_ increased with age; previous studies had suggested that older adults (≥ 65 years) are more susceptible to the health effects of air pollution compared to younger adults [35]. Perhaps due to cumulative effects of PM_2.5_ in human health, like toxicity, the lower immunity in these age-groups or the inflammation process related to this exposure might exacerbate the inflammation process resulting from aging [36]. However, the PAF calculated in this study is not age-specific and therefore, our results do not demonstrate this direct association.

Long-term exposure to PM_2.5_ was associated with mortality burden of IHD and stroke that were mainly concentrated in Lisbon Metropolitan Area, North and Centre. These areas, mainly the urban areas, are critical points of high pollutant emissions, characterised by dense population, significant emissions from human activity, transportation flow, and civil construction services, or industrial areas. Simultaneously, these are the regions with a higher number of deaths for each cause of death, so as expected, these regions are the ones with a higher mortality burden-PM_2.5_ exposure.

In our study, the higher mortality burden for each cause of death was observed in 2011 and this was consistent with the results of the GBD 2019 study [8]. From 2011 to 2019, the YLL rates we found in our study were slightly higher than those of the GBD 2019 study [8] and higher than the results of WHO estimates [37] for 2019 for Portugal, even without considering the autonomous regions. Differences in data sources may explain this gap. Our data were obtained from Statistics Portugal whereas the data for the GBD study were collected from the WHO Mortality Database [38] and the concentration–response function used in the GBD study was different than the one used in our study [39]. From the 2012 to 2021, our findings were higher than the results of the annual European Environment Agency (EEA) report [6, 16, 40–47], though in the EEA reports the results refer to all causes of death, while in our study the results only refer to IHD and stroke.

Between 2011 and 2021, the levels of PM_2.5_ decreased in Portugal and its different regions, and this trend is aligned with the trend of the levels of PM_2.5_ observed in Europe from 2005 to 2020 where a decrease of 32% of the emissions of this pollutant was observed [6]. The decreasing of the levels of exposure does translate into a decrease in the attributable health impacts from air pollution. As expected, the time trend of the attributable mortality burden of cardiovascular disease to PM_2.5_ exposure in Portugal follows the decreasing trend in exposure that occurred from 2011–2021. For the different regions of Portugal, mortality rates from IHD and stroke showed the major downward trend in Algarve and this trend follows the downward trend in PM_2.5_ levels, with the same region being the region with the greatest decrease. However, when observing the regions with a high density of population, these were the regions with a lower decrease of the mortality burden. These regions correspond to the regions with higher density of population and high sources of emission (e.g. traffic or industry), being important to implement policies that improve the air quality and, in this way, reduce the health impact in these hot spots.

The results for the different scenarios of exposure allowed us to estimate what is the impact on mortality burden attributable in those different scenarios compared to the current scenario. The worst scenario, with a concentration of PM_2.5_ being the annual highest hourly value registered in 2021 allowed us to estimate the impact of the exposure attributable to higher levels of PM_2.5_. The annual concentration of PM_2.5_ in 2019 and 2021, are similar and below the recommended value by WHO-AQG 2005. However, if we consider the levels recommended in WHO-AQG 2021, the estimated burden was lower compared to the current annual scenario. A previous study reported that premature deaths could be avoided annually by lowering air pollution concentrations, particularly below WHO guidelines [48]. In our study, we estimate the avoidable mortality burden if the annual levels of the exposure recommended, in 2021, by WHO (WHO-AQG 2021) would be adopted. As expected, the avoidable mortality burden for IHD and stroke is substantial, when WHO-AQG 2021 are considered. As EEA reported, in 2023, 97% of the European population is exposed to atmospheric levels of PM_2.5_ above the recommended levels of WHO-AQG 2021, where the Portuguese population is part of this percentage [49]. Reducing air pollution to these guideline levels would prevent a significant number of attributable deaths in EU [16]. No evidence indicates a safe level of exposure or a threshold below which no adverse health effects occur [50]. While the 2021 target recommended by WHO may be challenging, efforts must be made to achieve it to mitigate health effects, in Portugal. Our results provide an opportunity to reflect on the avoidable health impact, mainly in mortality, if the WHO-AQG 2021 was accomplished in Portugal. The results of the health impact attributable to the comparative analyse for pre-pandemic period (2019) and during the pandemic period (2021) allows us to conclude that the restrictions implemented in 2021 due to COVID-19, might had a positive effect in mortality burden attributable to PM_2.5_ exposure deriving from the decreased of the PM_2.5_ levels, probably related with the mobility restriction. For the attributable and avoidable burden, we performed a probabilistic approach to estimate the distribution of YLL rates. This method reinforces our findings and strengthens our results by providing results that reflect the natural variability of air pollution and therefore more accurate data on the impact on mortality attributed to exposure to the pollutant under study.

Cardiovascular disease has a significant burden amongst the Portuguese population, being the main cause of death. Our study provided a comprehensive and accurate health impact assessment of the impact of PM_2.5_ exposure in Portugal and shows that a significant share of the cardiovascular mortality burden is attributable to PM_2.5_.However, the exposure to PM_2.5_ is not only associated with premature mortality but also with morbidity due to several acute and chronic conditions, so it is important in future studies to estimate this important epidemiological parameter [6]. An additional important topic to estimate in further studies is the economic burden associated with this exposure. As the Organisation for Economic Co-operation and Development (OECD) states in a recent study, air pollution causes economy-wide reductions in market economic activity based on data for Europe, and the estimates show that a 1 μg/m^3^ increase in PM_2.5_ concentration causes a 0.8% reduction in gross domestic product (GDP) [51]. Since the health impact related to air pollution has economic costs, additional health expenditure, and reduced labour productivity, further studies to estimate the economic burden attributable to this exposure in Portugal are important.

Some limitations of this study should be pointed out, contributing for the uncertainty of the estimates. PM_2.5_ concentration data are not available for all regions of Portugal, and for our study to be representative of all regions during the period studied, it was necessary to include PM_10_ concentration data converted to PM_2.5_ concentration using the conversion factor described in the HRAPIE project [20]. The use of estimated PM_2.5_ data could contribute to an overestimation of exposure and consequently of the mortality burden. Also, the PAF not age and sex-specific could contribute to an overestimation of the mortality, especially when considering younger ages. The effects of air pollution on health do not depend only on exposure but also on the susceptibility of the population. The susceptibility to the impact of air pollution because of age, pre-existing health conditions or particular behaviours, such as diet or smoking. However, in our study, the previous morbidities that can contribute to increase the burden were not considered. The economic status of people exposed to this pollutant is another important point that should be considered since some clusters of the population has poorer health and less access to high-quality medical care, increasing their vulnerability to air pollution [52, 53].

## Conclusions

Our study reports significant findings about the mortality burden of cardiovascular disease attributable to PM_2.5_ exposure. Between 2011 and 2021, approximately 22% and 23% of IHD and stroke deaths were attributable to PM_2.5_ exposure. Nevertheless, the mortality burden attributable to cardiovascular diseases has been decreasing in last years in Portugal. This study also documents that if WHO recommended value of exposure (WHO-AQG 2021) were to be met in Portugal, a reduction on the mortality burden due to IHD and stroke attributable to PM_2.5_ exposure would be expected. These findings provide evidence of the impact of air pollution on human health, which are crucial for decision-making, at national and regional level. Further studies should therefore be carried out to estimate the total burden of cardiovascular disease, mortality, and morbidity, as well as the economic burden attributable to PM_2.5_ exposure, thus providing a broader view on the impact of air pollution in Portugal.

### Supplementary Information

**Supplementary Material 1. **

**Supplementary Material 2. **

**Supplementary Material 3. **

## Data Availability

The datasets used and/or analysed during the current study are available from the corresponding author on reasonable request.
