# Supplementary material for: Mortality burden of cardiovascular disease attributable to ambient PM2.5 exposure in Portugal, 2011 to 2021
Source: BMC Public Health. 2024 Apr 27;24:1188. doi: 10.1186/s12889-024-18572-0 (PMC11055300; doi:10.1186/s12889-024-18572-0)
Supplement: Supplementary file 1 — Supplementary Material 1. [file 12889_2024_18572_MOESM1_ESM.pdf]

Supplementary material

Table S1: Average of atmospheric levels of $PM_{2.5}$ (µg/m3), per year, for Portugal and its different regions, 2011-2021.

Year	Portugal	North	Centre	LMA*	Alentejo	Algarve
**2011**	16.5	16.3	15.7	18.2	11.9	14.6
**2012**	13.6	13.1	14.5	15.0	10.2	10.0
**2013**	13.2	12.5	13.7	15.1	12.4	10.7
**2014**	11.8	8.2	12.1	12.5	12.0	10.1
**2015**	12.9	11.5	12.0	14.6	10.8	13.1
**2016**	11.6	11.7	10.0	13.3	8.8	12.1
**2017**	12.7	11.3	11.0	14.4	11.3	13.4
**2018**	11.6	10.8	10.2	13.4	6.9	13.4
**2019**	10.5	9.2	10.0	11.9	5.9	13.1
**2020**	9.5	11.7	8.4	10.6	6.2	8.6
**2021**	9.6	11.4	10.1	10.6	7.2	5.7

*LMA: Lisbon Metropolitan Area

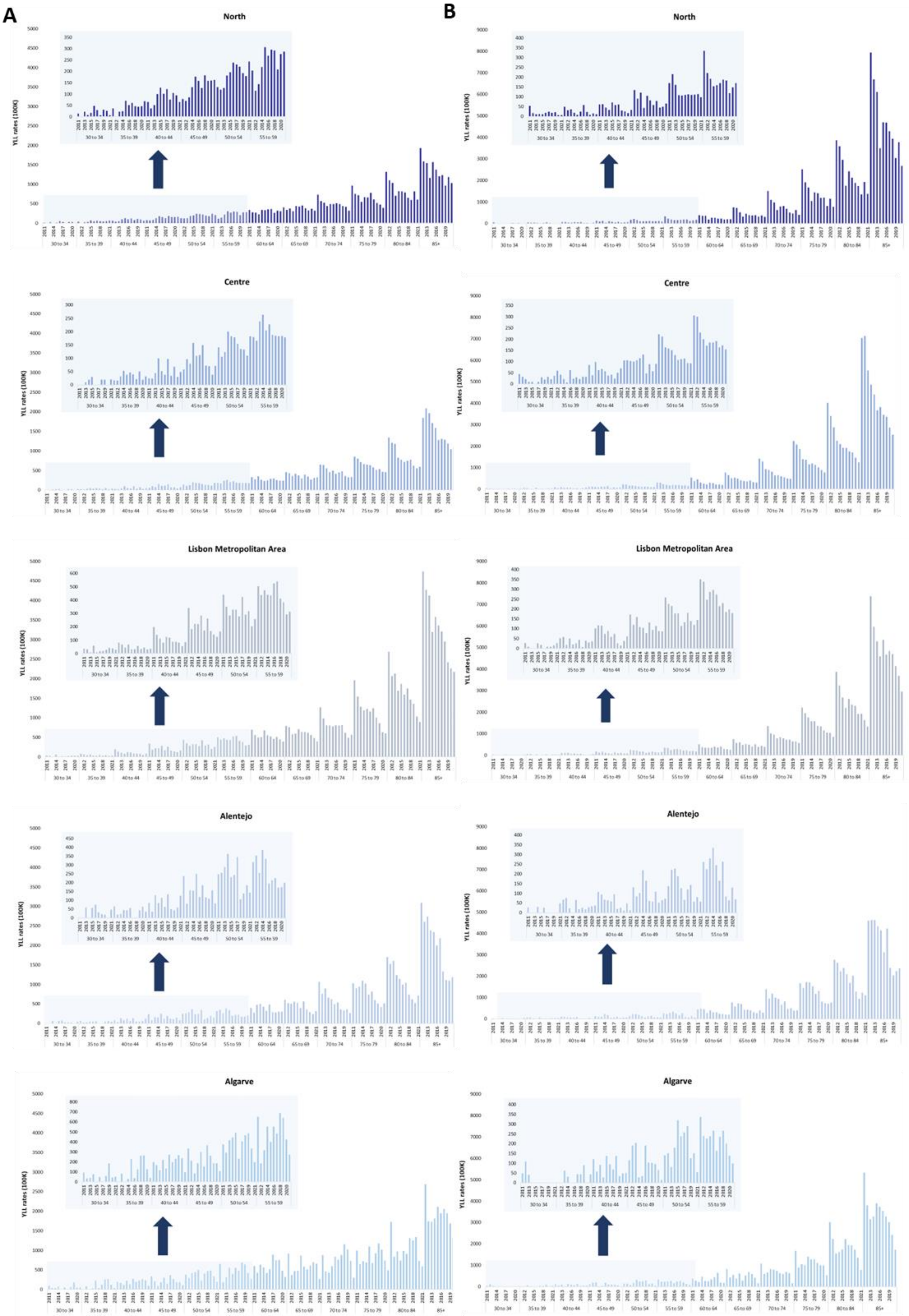

Figure S1: (A) IHD YLL rates (per 100K) and (B) Stroke YLL rates (per 100K) by age groups, 2011-

2021, for different regions of Portugal.

Table S4: Summary of the probabilistic approach performed, including the probabilistic distributions assigned to each variable.

**Cause of death**	**Input parameters**	**Notes**
**Ischemic Heart Disease**	$RR_{literature}$ = Triangular (1.15, 1.23, 1.31)	Attributable and avoidable burden calculation
	$[PM_{2.5}]_{2019}$ = Exponential (10.53)<br>$[PM_{2.5}]_{2021}$ = Exponential (9.63)	Attributable and avoidable burden calculation
	$[PM_{2.5}]_{WHO\text{-}AQG\ 2021}$ = Uniform (5)	Avoidable burden
**Stroke**	$RR_{literature}$ =Triangular (1.13, 1.24, 1.36)	Attributable and avoidable burden calculation
	$[PM_{2.5}]_{2019}$ = Exponential (10.53)<br>$[PM_{2.5}]_{2021}$ = Exponential (9.63)	Attributable and avoidable burden calculation
	$[PM_{2.5}]_{WHO\text{-}AQG\ 2021}$ = Uniform (5)	Avoidable burden

$[PM_{2.5}]$: $PM_{2.5}$ concentration

Table S5: Results of probabilistic approach for $PM_{2.5}$ exposure assessment, 2019 and 2021

**$[PM_{2.5}]$**	**Mean (P2.5 – P97.5)**	**Median (p50)**	**p75**
**2019**	10.5 (0.3 – 38.3)	7.3	14.6
**2021**	9.6 (0.2 - 35.5)	6.7	13.3

$[PM_{2.5}]$: $PM_{2.5}$ concentration; **P2.5:** Percentil 2.5; **P50:** Percentil 50; **P75:** Percentil 75; **P97.5:** Percentil 97.5

Table S6: Results of probabilistic approach of attributable mortality burden – $PM_{2.5}$ exposure, 2019 and 2021.

	**Cause of death**	**Mean (P2.5 – P97.5)**	**Median (p50)**	**p75**
**2019**				
**PAF (%)**	IHD	17.8 (0.5 – 55.9)	13.8	25.9
	Stroke	18.5 (0.6 – 57.9)	14.3	27.0
**YLL rates (per 100K)**	IHD	313.0 (9.5 – 980.9)	242.6	455.3
	Stroke	405.0 (12.3 – 1267.9)	313.9	590.0
**2021**				
**PAF (%)**	IHD	16.6 (0.5 – 52.5)	12.7	24.0
	Stroke	17.2 (0.5 – 54.8)	13.2	24.9
**YLL rates (per 100K)**	IHD	264.6 (7.9 – 839.2)	203.5	384.1
	Stroke	328.9 (9.8 – 1046.7)	252.5	476.3

PAF: Population attributable fraction; **YLL:** Years of life lost; **IHD:** ischemic heart disease*;* **P2.5:** Percentil 2.5; **P50:** Percentil 50; **P75:** Percentil 75; **P97.5:** Percentil 97.5

Table S7: Results of probabilistic approach of avoidable mortality burden – $PM_{2.5}$ exposure for different alternative scenarios, 2019 and 2021.

		**WHO AQG 2021**		
	**Cause of death**	**Mean (P2.5 – P97.5)**	**Median (p50)**	**p75**
**2019**				
**PIF (%)**	IHD	8.9 (-10.7 – 50.6)	4.6	17.9
	Stroke	9.2 (-11.6 – 52.5)	4.7	18.5
**YLL rates (per 100K)**	IHD	150.1 (-179.55 – 853.3)	77.2	301.0
	Stroke	201.7 (-254.1 – 1148.4)	103.2	404.2
**2021**				
**PIF (%)**	IHD	7.5 (-10.8 – 47.1)	3.4	15.7
	Stroke	7.8 (-11.7 – 49.1)	3.5	16.3
**YLL rates (per 100K)**	IHD	119.7 (-172.0 – 751.9)	53.6	251.5
	Stroke	148.2 (-224.2 – 938.6)	66.2	311.6

PIF: Potential impact fraction; **YLL:** Years of life lost; **IHD:** ischemic heart disease; **P2.5:** Percentil 2.5; **P97.5:** Percentil 97.5; **P50:** Percentil 50; **P75:** Percentil 75
