# Supplementary material for: Mortality burden of cardiovascular disease attributable to ambient PM2.5 exposure in Portugal, 2011 to 2021
Source: BMC Public Health. 2024 Apr 27;24:1188. doi: 10.1186/s12889-024-18572-0 (PMC11055300; doi:10.1186/s12889-024-18572-0)
Supplement: Supplementary file 2 — Supplementary Material 2. [file 12889_2024_18572_MOESM2_ESM.pdf]

Table S2: Years of life lost (YLL) and years of life lost rate per 100K (YLL rate) related to environmental burden of disease, by different years (2011-2021), age-groups, and sexes for Portugal and its different regions for ischemic heart disease.

Year	Age group	Portugal				North				Centre				Lisbon Metropolitan Area				Alentejo				Algarve			
		Males		Females		Males		Females		Males		Females		Males		Females		Males		Females		Males		Females	
		YLL (EBD)	YLL rate (EBD)	YLL (EBD)	YLL rate (EBD)	YLL (EBD)	YLL rate (EBD)	YLL (EBD)	YLL rate (EBD)	YLL (EBD)	YLL rate (EBD)	YLL (EBD)	YLL rate (EBD)	YLL (EBD)	YLL rate (EBD)	YLL (EBD)	YLL rate (EBD)	YLL (EBD)	YLL rate (EBD)	YLL (EBD)	YLL rate (EBD)	YLL (EBD)	YLL rate (EBD)	YLL (EBD)	YLL rate (EBD)
	30 - 34	119.7	34.6	17.1	4.7	34.0	26.9	0.0	0.0	0.0	0.0	0.0	0.0	55.9	54.7	18.6	17.0	0.0	0.0	0.0	0.0	30.8	192.2	0.0	0.0
	35 - 39	266.4	69.4	94.0	23.2	93.4	65.7	15.6	10.3	30.1	36.2	0.0	0.0	136.5	120.9	51.2	42.5	11.8	43.1	23.7	86.6	0.0	0.0	0.0	0.0
	40 - 44	555.5	153.7	170.9	45.0	184.0	132.8	0.0	0.0	41.1	50.4	0.0	0.0	279.2	281.2	124.1	118.6	21.5	82.8	21.5	84.4	38.5	242.3	25.7	153.3
	45 - 49	962.4	269.4	256.6	67.2	204.0	145.0	25.5	16.9	98.7	119.9	0.0	0.0	489.1	529.2	167.7	166.7	77.6	292.0	48.5	181.7	92.6	607.6	11.6	72.3
	50 - 54	1282.0	379.1	354.8	97.7	273.0	208.7	79.6	56.9	187.1	234.1	44.0	52.2	598.4	692.3	211.9	217.9	103.8	396.2	26.0	98.8	113.5	764.1	0.0	0.0
	55 - 59	1673.8	545.0	332.8	97.9	420.8	367.1	70.1	55.4	242.4	331.4	29.1	37.2	735.8	898.5	164.7	170.8	122.0	510.8	30.5	125.0	145.5	1072.6	36.4	254.9
2011	60 - 64	2131.9	743.7	664.0	205.6	581.7	572.0	156.3	138.0	327.6	492.5	134.4	181.6	961.1	1148.5	304.5	308.5	138.7	634.8	39.6	167.5	126.1	977.4	31.5	236.2
	65 - 69	2306.6	962.9	749.1	259.2	493.8	619.6	213.7	221.1	449.3	787.7	135.5	194.1	969.3	1319.3	306.9	350.5	207.5	1109.6	44.9	196.1	160.5	1506.6	46.8	388.8
	70 - 74	2809.6	1368.0	1438.6	552.1	702.5	1058.7	409.3	477.6	537.9	1009.2	248.3	367.0	1064.6	1845.7	582.5	806.3	311.4	1696.8	139.4	582.7	138.6	1430.4	44.4	402.0
	75 - 79	2835.3	1627.0	2237.6	912.9	736.2	1320.5	564.4	710.4	569.9	1193.3	403.7	612.2	1145.8	2571.9	995.2	1543.4	246.5	1362.5	190.5	779.6	111.4	1373.6	75.7	701.5
	80 - 84	2511.9	2245.8	2653.4	1459.6	615.4	1744.6	615.4	1058.1	577.2	1819.2	518.3	1035.0	911.9	3321.6	1115.9	2331.2	248.6	2087.8	257.3	1444.3	113.8	2073.2	117.2	1490.5
	≥ 85	2452.4	3332.7	4050.7	2587.1	619.2	2818.7	731.0	1521.3	488.2	2287.0	726.8	1627.0	933.2	5202.5	1894.7	4541.8	246.5	2965.8	469.0	3162.4	124.9	3106.6	179.5	2458.5
	**Total**	19907.3	625.2	13019.7	352.9	4958.0	429.6	2881.0	216.6	3549.5	471.0	2240.1	256.3	8280.7	941.1	5938.0	570.1	1736.0	687.5	1290.8	456.8	1196.2	828.3	568.8	354.9
	30 - 34	43.4	13.2	29.0	8.3	0.0	0.0	0.0	0.0	0.0	0.0	0.0	0.0	31.6	32.8	31.6	30.2	0.0	0.0	0.0	0.0	11.0	72.5	0.0	0.0
	35 - 39	212.3	55.8	13.3	3.3	0.0	0.0	0.0	0.0	28.1	34.2	0.0	0.0	144.6	128.1	14.5	11.9	10.3	37.2	0.0	0.0	30.3	168.1	0.0	0.0
	40 - 44	410.1	113.1	96.5	25.2	105.4	76.4	0.0	0.0	25.6	31.4	12.8	14.9	197.2	194.8	92.0	85.7	18.7	71.4	0.0	0.0	55.1	346.6	0.0	0.0
	45 - 49	630.4	178.2	195.6	51.4	137.2	98.8	63.3	42.1	115.3	142.0	46.1	53.3	308.0	332.2	47.4	46.9	25.3	97.4	16.9	64.1	49.7	327.1	16.6	102.0
	50 - 54	1163.4	341.8	223.0	60.7	291.8	220.2	37.6	26.4	144.0	179.1	30.8	36.0	560.1	650.3	84.5	87.0	112.8	426.0	22.6	84.8	51.7	347.5	36.9	237.1
	55 - 59	1195.6	386.3	333.1	97.5	223.9	191.8	58.0	45.3	208.3	282.3	45.3	57.3	577.2	709.4	195.5	205.5	139.1	580.6	33.1	133.3	45.5	332.1	6.5	45.2
2012	60 - 64	1850.0	633.1	414.4	125.8	502.9	477.9	114.9	97.6	306.1	445.5	94.2	124.5	855.0	1032.0	169.4	171.3	86.1	384.1	40.2	170.1	112.7	865.5	0.0	0.0
	65 - 69	1928.5	790.3	766.4	260.4	469.6	574.9	146.4	148.3	379.8	660.2	173.2	247.3	883.3	1180.9	362.9	402.8	151.1	789.0	68.2	295.3	62.2	572.1	23.9	193.0
	70 - 74	2244.3	1099.1	1062.3	411.1	581.4	879.8	273.0	322.6	541.2	1035.4	220.9	330.4	885.4	1511.3	408.7	559.8	177.7	1015.5	129.3	561.2	71.4	732.4	23.8	218.2
	75 - 79	2342.8	1342.6	1769.7	722.5	568.6	1010.8	454.9	568.4	492.5	1039.7	417.1	637.8	989.6	2191.6	706.8	1095.7	211.0	1191.5	165.5	683.4	86.0	1072.6	35.1	324.5
	80 - 84	2153.4	1847.4	2150.2	1144.0	553.3	1501.6	512.4	850.3	532.3	1626.1	480.8	935.7	751.8	2597.1	878.9	1764.4	236.1	1917.0	231.1	1259.1	66.6	1165.5	49.3	607.5
	≥ 85	2167.5	2866.4	3838.9	2377.4	453.1	1996.8	699.8	1407.9	546.9	2508.3	852.7	1876.0	863.0	4643.3	1800.6	4119.6	238.9	2817.7	375.3	2482.6	65.1	1604.6	135.9	1814.1
	**Total**	16341.8	513.4	10892.2	294.2	3887.0	336.4	2360.4	176.4	3320.1	441.5	2374.0	271.3	7046.9	801.2	4792.8	457.9	1407.1	558.9	1082.2	383.8	707.5	490.1	328.0	203.5
	30 - 34	113.4	35.7	14.2	4.2	54.0	46.0	0.0	0.0	14.6	20.9	0.0	0.0	15.9	17.2	0.0	0.0	13.4	57.7	13.4	58.9	11.8	80.6	0.0	0.0
	35 - 39	181.8	49.0	39.0	9.8	49.5	36.5	12.4	8.5	40.1	49.8	13.4	15.5	87.2	79.1	14.5	12.1	12.3	45.3	0.0	0.0	0.0	0.0	0.0	0.0
	40 - 44	460.5	127.2	94.5	24.4	146.1	106.8	0.0	0.0	24.3	30.1	48.6	56.6	198.2	193.6	39.6	35.9	67.1	256.2	0.0	0.0	29.4	183.9	9.8	56.9
	45 - 49	765.9	218.9	117.0	30.9	222.8	163.3	20.3	13.6	109.4	136.5	21.9	25.5	357.2	386.6	71.4	70.8	70.5	274.7	10.1	38.9	26.5	174.5	0.0	0.0
	50 - 54	1129.2	331.3	218.3	58.9	289.1	216.4	63.2	43.6	175.6	218.9	29.3	33.9	446.1	518.5	74.3	76.7	125.8	476.9	27.0	101.4	102.5	705.5	23.7	151.0
	55 - 59	1412.7	451.8	309.3	89.4	302.4	255.0	55.7	42.6	309.4	413.2	60.2	75.1	683.0	845.4	149.7	157.6	110.8	453.5	15.8	62.5	55.6	399.0	34.7	237.5
2013	60 - 64	1818.1	626.5	536.0	163.6	462.0	438.0	137.9	116.8	417.1	606.0	96.8	128.1	656.7	817.0	227.0	234.6	171.5	759.9	48.0	202.9	132.4	1024.3	36.1	265.7
	65 - 69	1733.9	688.7	700.9	231.2	491.7	573.7	216.6	210.2	373.0	630.5	94.8	132.5	667.5	877.8	309.7	334.6	151.4	773.4	64.1	275.7	81.7	725.5	30.7	241.0
	70 - 74	2013.2	985.6	907.2	350.7	582.2	881.0	208.6	247.1	440.2	858.3	209.6	317.5	701.7	1166.7	393.6	526.6	251.0	1475.1	101.4	450.4	67.8	695.8	21.2	192.3
	75 - 79	2133.4	1210.7	1523.3	622.4	627.6	1099.2	358.6	445.3	425.3	893.2	374.8	577.0	802.1	1740.3	618.7	954.4	225.0	1299.0	162.9	686.0	88.5	1091.6	47.6	445.5
	80 - 84	2196.5	1822.9	2130.0	1102.6	525.0	1377.2	506.9	820.7	544.3	1620.4	475.9	901.2	897.5	2970.3	837.2	1631.4	237.2	1885.1	267.2	1411.8	52.7	879.4	86.9	1038.8
	≥ 85	2198.1	2807.7	3700.2	2204.3	446.3	1884.0	719.5	1387.7	558.3	2490.2	804.3	1715.5	907.5	4697.1	1777.6	3880.0	251.5	2879.2	414.6	2657.4	91.4	2215.9	113.3	1468.1
	**Total**	16156.9	508.9	10289.8	277.2	4198.5	363.7	2299.7	171.2	3431.7	458.2	2229.4	254.8	6420.6	732.7	4513.5	429.8	1687.6	672.6	1124.4	399.6	740.3	514.0	404.0	249.6
	30 - 34	153.6	50.1	25.6	7.9	18.4	16.2	0.0	0.0	26.3	39.1	0.0	0.0	108.3	121.4	0.0	0.0	0.0	0.0	0.0	0.0	0.0	0.0	22.4	149.6
	35 - 39	246.3	68.7	105.6	27.3	50.7	39.0	16.9	12.0	60.3	77.2	24.1	28.7	111.7	104.0	37.2	31.6	11.9	45.6	11.9	44.9	0.0	0.0	10.3	57.0
	40 - 44	725.1	199.6	128.0	32.5	261.0	192.2	23.0	15.4	131.6	164.6	32.9	38.1	135.3	128.7	45.1	39.5	43.3	167.0	0.0	0.0	56.1	342.3	18.7	105.6
	45 - 49	1076.0	312.2	230.6	61.5	290.5	218.7	76.1	51.5	207.5	262.7	49.4	58.5	365.8	395.9	61.0	60.5	68.2	270.4	9.7	38.3	50.5	332.3	8.4	51.9
	50 - 54	1653.9	482.6	299.9	79.8	413.4	306.9	98.7	66.7	282.0	351.5	52.9	60.5	525.7	604.7	81.6	83.3	165.2	629.5	26.1	98.1	127.7	877.0	7.5	47.3
	55 - 59	1932.6	614.7	339.7	97.3	494.6	412.7	59.8	45.0	357.1	474.5	54.3	67.0	638.8	790.3	135.7	142.6	145.5	594.8	46.0	180.8	132.3	947.1	26.5	179.7
2014	60 - 64	1910.0	658.2	392.5	119.0	433.3	406.2	75.4	62.5	316.2	456.1	60.5	79.9	719.6	913.7	159.1	166.0	199.1	879.5	33.2	139.2	108.9	849.3	40.1	291.6
	65 - 69	1937.8	751.0	744.0	242.3	439.8	492.5	147.9	140.4	399.7	659.7	148.5	205.9	740.0	963.8	281.9	300.8	152.1	767.6	84.5	367.0	77.9	673.8	38.9	304.7
	70 - 74	1960.8	950.2	934.4	357.5	467.3	696.6	198.8	232.4	355.0	701.6	175.2	267.4	744.9	1194.8	374.9	487.3	163.4	983.3	93.4	422.3	88.7	915.1	36.3	323.3
	75 - 79	2170.7	1223.9	1457.0	592.5	476.6	830.1	279.6	344.7	433.6	914.7	308.1	474.6	770.6	1625.9	578.9	878.6	258.9	1526.7	180.1	770.6	90.7	1106.7	38.9	365.9
	80 - 84	1809.0	1451.2	1771.8	894.4	362.7	914.1	362.7	572.2	376.8	1079.6	362.1	667.7	684.3	2205.2	726.7	1391.9	229.4	1780.8	171.3	877.4	57.7	935.9	52.7	601.9
	≥ 85	1972.1	2441.9	3363.1	1935.5	390.3	1587.4	525.6	972.2	426.5	1858.1	786.4	1639.0	722.0	3598.3	1439.4	3020.8	227.9	2540.2	370.3	2293.3	94.7	2265.0	124.9	1578.6
	**Total**	17548.0	554.0	9792.2	263.1	4098.6	355.9	1864.5	138.2	3372.8	452.2	2054.4	235.0	6267.1	713.6	3921.6	371.6	1664.9	671.2	1026.5	366.7	885.2	615.5	425.7	261.8
	30 - 34	111.3	37.9	13.9	4.5	37.8	34.5	0.0	0.0	26.1	40.3	13.0	19.3	15.4	18.1	0.0	0.0	23.8	112.8	0.0	0.0	0.0	0.0	0.0	0.0
	35 - 39	382.6	110.7	51.0	13.6	184.5	147.7	0.0	0.0	35.9	47.0	23.9	29.2	70.7	68.7	0.0	0.0	21.8	87.0	0.0	0.0	51.5	314.5	25.7	146.1
	40 - 44	765.2	208.6	104.3	26.1	325.0	239.7	41.9	27.9	76.1	94.7	10.9	12.4	257.0	238.5	25.7	21.8	59.5	227.9	0.0	0.0	23.4	138.0	23.4	128.5
	45 - 49	1211.7	353.0	261.1	69.8	434.5	330.8	56.7	38.8	146.8	187.1	29.4	34.8	463.2	496.8	92.6	90.7	98.3	392.6	26.8	106.0	42.1	277.1	52.7	319.6

2015	50 - 54	1500.1	438.6	279.5	74.0	480.2	356.6	75.8	50.7	288.2	360.0	17.5	20.0	485.5	557.1	123.9	125.8	95.6	371.9	23.9	91.1	112.8	781.0	37.6	236.5
	55 - 59	1838.6	577.7	385.8	109.3	690.2	567.5	96.5	71.7	261.5	343.2	61.5	74.9	591.4	724.2	182.0	190.0	140.4	567.1	28.1	109.8	99.4	709.6	16.6	110.5
	60 - 64	2005.6	688.4	640.1	192.1	617.4	571.2	173.6	141.1	273.3	392.3	66.7	86.6	820.0	1055.2	354.8	373.5	164.2	718.3	36.5	151.5	100.4	771.6	14.3	101.5
	65 - 69	2239.8	852.8	827.1	267.5	611.4	666.6	256.6	240.2	413.0	669.9	84.9	118.2	896.8	1156.4	321.2	338.9	134.3	672.0	92.9	405.8	158.3	1350.7	54.8	426.5
	70 - 74	2106.9	996.2	835.7	310.4	574.7	827.4	199.1	222.9	394.0	769.5	182.9	275.2	865.5	1338.6	266.3	331.7	137.0	829.4	98.4	452.7	111.1	1148.2	65.6	576.7
	75 - 79	2243.6	1270.8	1463.5	602.7	567.2	990.7	349.0	436.9	433.3	932.6	278.9	437.5	829.0	1722.7	570.5	865.2	227.0	1382.5	171.9	755.6	142.0	1714.3	64.9	619.3
	80 - 84	1948.2	1521.7	1789.5	887.1	444.6	1082.1	427.8	659.6	393.8	1101.6	309.2	563.4	797.0	2495.3	793.5	1494.4	191.7	1464.8	183.7	926.9	94.2	1526.0	56.5	623.0
	≥ 85	2165.3	2556.9	3790.5	2090.1	492.7	1901.6	802.7	1418.8	391.8	1629.4	785.8	1571.4	898.2	4254.7	1642.8	3298.9	247.1	2685.8	361.6	2151.1	104.2	2357.8	161.1	1972.8
	**Total**	18518.8	585.2	10442.1	280.1	5460.2	474.3	2479.7	183.3	3133.8	420.7	1864.4	213.3	6989.7	795.1	4373.4	412.4	1540.6	626.6	1023.9	368.8	1039.4	723.8	573.3	350.2
2016	30 - 34	139.4	49.3	38.0	12.8	76.5	72.5	25.5	23.1	0.0	0.0	0.0	0.0	28.5	34.7	0.0	0.0	29.6	145.7	0.0	0.0	0.0	0.0	13.1	95.7
	35 - 39	290.3	87.6	23.2	6.4	116.9	97.7	11.7	8.9	70.8	96.4	0.0	0.0	65.3	66.2	0.0	0.0	18.1	75.2	9.0	37.2	12.0	77.1	0.0	0.0
	40 - 44	580.6	157.8	158.3	39.3	201.9	150.0	85.0	57.1	55.2	68.7	9.2	10.5	201.9	184.8	71.3	59.0	32.9	125.1	0.0	0.0	98.5	568.1	0.0	0.0
	45 - 49	941.6	273.2	133.2	35.3	402.0	307.0	38.3	26.2	174.0	222.0	8.3	9.8	267.5	281.0	74.9	71.8	51.8	207.8	7.4	29.5	39.4	259.3	9.9	58.8
	50 - 54	1433.8	420.4	271.5	71.8	589.2	442.0	85.4	57.2	251.2	317.1	44.3	51.1	429.5	484.9	95.4	95.4	105.6	415.8	19.8	75.7	44.0	302.9	26.4	165.7
	55 - 59	1853.5	575.3	381.2	106.2	594.2	478.4	112.8	81.7	286.4	371.9	78.1	93.2	798.8	977.6	134.5	140.1	75.6	301.0	23.3	89.9	131.7	932.8	31.0	203.0
	60 - 64	1910.4	656.0	459.8	137.5	606.1	558.2	176.0	141.4	293.3	418.2	73.3	94.8	765.0	994.1	182.1	192.9	136.1	599.8	15.1	62.8	120.9	939.2	20.1	142.1
	65 - 69	2050.4	762.1	632.2	200.7	630.7	660.9	243.4	220.7	320.8	509.7	81.4	112.2	859.6	1102.4	253.6	264.6	136.9	666.4	34.2	148.3	119.7	984.5	28.5	216.3
	70 - 74	1964.0	907.6	879.5	319.0	577.9	802.6	229.3	249.2	313.5	605.5	182.6	272.9	866.4	1304.9	343.5	411.2	127.7	765.8	60.3	277.7	99.2	1039.0	61.4	531.1
	75 - 79	2046.5	1177.9	1281.4	537.3	556.4	991.5	335.3	428.2	440.0	974.0	239.2	383.7	790.8	1618.9	547.8	830.1	156.7	1022.3	116.8	539.6	87.3	1056.0	38.0	371.8
	80 - 84	1813.6	1390.5	1615.3	786.8	493.4	1175.0	390.7	588.1	355.4	991.2	301.1	542.6	695.0	2089.7	698.2	1289.6	172.0	1300.0	158.8	791.5	79.3	1302.6	58.8	642.1
	≥ 85	2067.9	2312.2	3398.0	1802.4	501.5	1798.3	695.2	1179.2	383.7	1514.2	607.2	1166.4	846.9	3840.9	1628.7	3157.0	221.4	2322.0	317.9	1822.5	95.3	2060.9	159.6	1881.3
	**Total**	17091.9	540.8	9271.4	248.4	5346.6	464.9	2428.6	179.3	2944.3	396.8	1624.7	186.2	6615.2	750.9	4030.1	378.2	1264.4	518.0	762.7	276.4	927.4	648.1	446.8	272.4
2017	30 - 34	82.3	30.2	27.4	9.6	49.6	48.6	12.4	11.7	0.0	0.0	0.0	0.0	30.5	38.6	0.0	0.0	0.0	0.0	12.4	66.7	0.0	0.0	0.0	0.0
	35 - 39	301.5	94.6	62.8	18.0	147.6	127.5	0.0	0.0	44.2	64.1	11.0	15.1	69.9	74.1	41.9	39.6	0.0	0.0	0.0	0.0	26.2	176.4	13.1	80.4
	40 - 44	776.6	211.3	114.2	28.3	289.1	217.4	51.6	35.1	150.5	191.0	10.0	11.7	165.2	149.7	38.1	31.2	61.8	232.4	10.3	37.9	71.5	407.4	0.0	0.0
	45 - 49	1111.3	319.5	257.3	67.4	288.3	220.1	65.1	44.3	208.0	265.6	36.2	42.3	423.6	432.4	114.5	106.4	74.2	295.1	18.5	74.1	96.6	633.4	21.5	127.0
	50 - 54	1716.4	506.9	321.3	85.1	555.9	422.1	91.3	61.4	201.6	260.7	48.4	56.9	663.8	741.7	143.0	141.6	157.2	635.0	16.5	64.3	105.3	724.6	19.1	118.6
	55 - 59	2021.4	622.8	323.4	89.0	672.4	534.3	116.9	83.1	248.7	321.7	56.8	66.4	845.6	1032.4	116.9	121.3	94.7	373.9	14.6	55.8	134.9	955.7	8.4	54.5
	60 - 64	2199.9	748.3	518.5	153.9	671.3	607.2	171.0	135.4	369.4	515.7	61.6	77.8	670.4	869.5	233.8	249.0	221.0	972.5	6.3	25.9	197.3	1522.0	43.8	308.2
	65 - 69	2402.7	875.5	689.9	214.4	790.3	801.3	177.4	154.6	428.5	661.8	115.0	155.8	820.5	1052.7	297.8	308.2	171.5	819.2	75.0	326.4	117.8	966.5	12.4	93.0
	70 - 74	2110.0	952.7	995.9	353.8	557.0	751.4	298.6	317.1	424.6	791.6	130.0	189.9	789.8	1156.7	449.8	520.4	146.6	856.4	62.2	284.0	144.0	1477.1	46.3	388.9
	75 - 79	2111.2	1216.8	1524.9	643.0	601.5	1071.8	444.0	572.6	351.5	810.2	247.1	407.1	854.9	1703.7	608.1	907.7	160.6	1100.0	128.5	614.3	103.3	1249.0	66.1	658.4
	80 - 84	1940.7	1477.3	1744.5	845.3	493.3	1160.8	368.6	547.1	369.1	1033.3	307.1	558.2	764.1	2243.0	781.1	1436.2	174.1	1328.4	171.3	853.3	99.1	1662.2	99.1	1081.2
	≥ 85	2209.5	2365.1	3405.6	1737.0	489.4	1673.8	604.4	980.0	441.2	1602.4	652.6	1165.5	836.5	3614.8	1629.3	3029.6	273.2	2781.4	337.8	1856.7	115.8	2428.9	161.7	1855.7
	**Total**	18983.6	601.1	9985.6	267.1	5605.7	487.3	2401.2	176.6	3237.3	439.9	1675.8	193.1	6934.7	784.8	4454.4	415.9	1535.0	632.5	853.5	311.4	1211.7	851.8	491.5	299.9
2018	30 - 34	63.3	23.8	38.0	13.7	11.9	11.9	0.0	0.0	11.3	19.3	11.3	19.0	43.0	56.1	0.0	0.0	0.0	0.0	7.9	43.9	0.0	0.0	14.3	118.4
	35 - 39	266.9	85.9	11.6	3.4	98.1	86.7	10.9	8.8	31.1	45.1	0.0	0.0	65.7	71.6	0.0	0.0	0.0	0.0	0.0	0.0	78.7	544.8	0.0	0.0
	40 - 44	485.2	134.2	94.9	23.8	188.3	144.2	19.8	13.7	56.5	71.7	0.0	0.0	143.4	131.9	59.8	49.0	19.6	75.4	6.5	24.4	71.6	412.7	11.9	64.0
	45 - 49	950.2	271.4	209.1	54.0	410.7	313.6	98.2	66.4	93.4	119.2	25.5	29.8	290.8	290.1	64.6	58.0	53.1	211.7	5.9	23.3	75.2	483.2	10.7	62.2
	50 - 54	1466.4	435.6	135.6	36.0	573.4	440.8	39.8	26.9	204.4	264.3	15.1	17.8	480.3	532.8	76.8	75.6	52.6	215.9	0.0	0.0	134.2	916.4	9.6	59.6
	55 - 59	1777.1	545.0	418.1	113.8	638.4	501.6	154.3	107.6	246.7	319.2	53.3	62.3	575.4	697.9	160.8	166.0	97.4	386.4	18.5	71.1	177.4	1278.4	25.3	162.7
	60 - 64	2025.1	680.1	511.1	149.5	711.2	631.9	164.1	127.2	358.3	500.2	86.7	109.6	652.6	844.5	146.6	155.1	104.4	451.3	32.1	129.4	139.0	1054.0	65.9	456.8
	65 - 69	2054.0	752.0	692.0	215.8	624.3	631.1	201.2	174.5	377.7	583.2	103.0	139.6	728.2	952.7	336.1	352.5	133.0	633.9	23.9	104.1	124.2	1031.3	31.1	233.0
	70 - 74	2203.5	962.2	965.2	332.3	581.7	747.3	282.3	286.4	426.9	796.0	154.5	225.7	908.1	1298.5	387.0	433.6	90.4	517.2	50.9	231.0	180.2	1780.2	77.2	634.8
	75 - 79	1898.3	1091.3	1174.1	493.7	512.0	909.0	305.8	394.6	326.7	752.8	212.3	349.8	754.4	1452.3	464.3	672.1	124.9	884.4	81.8	400.0	136.5	1674.1	78.6	778.3
	80 - 84	1812.0	1360.7	1469.4	710.9	407.0	938.0	321.8	472.8	381.8	1068.9	321.1	583.6	728.3	2065.4	593.6	1081.8	128.3	1000.8	110.7	563.3	115.3	1931.7	73.7	812.4
	≥ 85	2055.3	2103.4	3345.7	1640.6	473.7	1532.0	702.5	1091.8	427.4	1552.4	648.7	1158.6	847.4	3508.5	1503.6	2709.7	139.3	1374.7	250.7	1313.6	113.6	2282.4	159.5	1768.0
	**Total**	17057.1	540.6	9064.8	241.9	5230.5	454.2	2300.9	168.5	2942.1	399.8	1631.6	188.0	6217.8	702.5	3793.2	352.2	943.1	392.1	588.9	216.0	1346.0	947.6	557.8	341.1
2019	30 - 34	173.1	66.1	23.1	8.5	51.3	52.0	10.3	10.0	22.1	37.7	0.0	0.0	64.5	86.3	0.0	0.0	6.8	36.5	0.0	0.0	28.0	248.9	14.0	120.1
	35 - 39	317.3	104.0	42.3	12.8	94.1	84.3	9.4	7.7	70.7	103.9	0.0	0.0	70.9	79.1	11.8	11.7	12.4	57.0	6.2	28.6	64.2	459.8	12.8	83.9
	40 - 44	596.1	168.3	115.4	29.6	230.9	181.2	51.3	36.4	82.7	105.8	27.6	32.6	161.1	151.9	21.5	18.0	16.9	65.9	5.6	21.4	93.4	555.7	0.0	0.0
	45 - 49	883.6	248.6	164.6	41.6	377.5	286.5	69.3	46.5	115.9	147.2	0.0	0.0	241.9	233.2	67.7	58.3	45.8	181.4	10.2	39.3	52.6	329.0	10.5	59.3
	50 - 54	1375.4	411.2	231.8	62.0	433.0	338.5	96.2	65.6	184.6	239.5	29.5	35.1	526.5	581.6	86.3	84.6	63.6	261.4	4.5	18.1	140.8	959.3	9.4	58.1
	55 - 59	1531.5	464.2	360.8	96.5	460.1	356.1	115.0	78.4	266.7	343.5	32.5	37.5	562.6	671.2	136.9	139.1	80.0	316.1	8.0	30.6	115.8	831.3	74.4	471.9
	60 - 64	1893.3	629.5	430.6	124.3	524.6	458.9	120.7	92.0	326.9	451.7	56.4	70.2	737.9	950.4	151.5	158.9	97.0	417.5	38.1	152.6	164.8	1249.5	43.0	295.7
	65 - 69	1822.4	665.5	585.8	180.9	516.6	515.2	195.9	166.1	325.3	498.1	47.8	64.5	704.6	935.9	251.7	264.7	94.1	447.4	32.4	139.6	133.8	1119.5	42.6	315.9
	70 - 74	1776.3	754.5	896.5	304.0	538.9	663.0	262.1	259.4	285.5	518.8	162.6	235.4	653.7	921.3	343.1	378.2	80.5	453.3	48.8	223.5	181.5	1744.5	50.4	411.9
	75 - 79	1730.6	981.2	1060.3	440.2	415.2	722.4	293.6	372.8	372.8	868.6	178.4	295.9	674.1	1247.1	413.4	579.2	94.0	680.0	76.4	379.5	133.7	1652.3	52.7	511.4
	80 - 84	1662.1	1238.2	1306.0	627.9	404.0	924.2	264.0	384.6	303.3	854.0	261.4	475.0	680.4	1865.3	576.6	1029.4	109.2	874.3	88.0	454.8	116.0	1919.1	84.7	944.8
	≥ 85	1883.4	1838.7	2833.8	1342.5	407.1	1247.5	554.3	827.4	422.4	1466.7	604.8	1045.4	750.6	2966.6	1237.9	2176.3	139.6	1320.8	200.3	1006.5	89.9	1754.0	156.2	1650.5
	**Total**	15645.1	494.5	8050.8	214.1	4453.4	385.1	2042.2	148.8	2778.7	376.4	1401.0	161.4	5828.7	656.2	3298.3	304.3	840.0	350.2	518.5	190.4	1314.6	929.3	550.7	336.1
	30 - 34	63.1	24.1	42.1	15.5	38.2	38.7	12.7	12.5	0.0	0.0	0.0	0.0	35.1	47.3	23.4	29.1	0.0	0.0	0.0	0.0	0.0	0.0	9.7	83.6
	35 - 39	192.9	65.2	48.2	15.1	81.7	75.2	23.4	19.9	26.0	39.2	0.0	0.0	42.9	49.4	10.7	11.1	26.2	125.3	0.0	0.0	17.7	134.1	17.7	120.0
	40 - 44	429.6	124.7	61.4	16.2	212.3	171.5	31.8	23.2	47.3	61.1	0.0	0.0	107.2	105.0	9.7	8.4	23.9	95.4	6.0	23.2	64.4	396.5	16.1	90.4
	45 - 49	711.0	197.0	173.8	43.2	344.3	260.6	105.2	70.0	49.8	62.5	14.2	16.2	254.7	238.1	26.3	22.0	69.8	272.8	10.7	40.9	36.2	218.5	29.0	159.1

	50 - 54	1000.6	299.3	204.4	54.6	452.1	356.5	34.1	23.5	120.5	157.0	57.1	67.7	360.4	392.4	39.2	37.9	71.9	296.4	19.2	76.6	58.2	395.7	45.3	276.2
	55 - 59	1427.8	433.2	291.8	77.5	653.8	506.2	112.7	75.9	245.8	316.9	55.9	64.0	427.9	507.9	110.4	111.4	76.0	306.1	12.7	48.9	108.2	784.6	17.1	108.3
2020	60 - 64	1742.7	571.9	381.9	108.9	625.1	538.8	162.8	122.3	309.8	422.4	58.1	71.1	657.8	836.7	125.6	130.9	109.7	466.1	36.6	145.4	118.5	897.5	14.8	99.9
	65 - 69	1757.9	639.8	470.3	143.6	668.8	658.8	143.7	119.3	324.6	493.8	110.9	146.9	629.4	848.4	182.7	194.0	77.6	365.1	27.9	119.2	150.8	1251.2	21.0	151.7
	70 - 74	1570.7	656.3	635.9	214.1	577.3	692.3	201.6	197.0	330.4	590.5	71.5	103.8	547.0	762.8	256.7	279.6	77.2	432.6	66.9	308.4	111.1	1065.5	55.6	452.3
	75 - 79	1328.9	734.7	869.7	350.9	430.7	723.7	246.6	299.9	287.3	661.5	197.0	322.4	493.6	879.3	334.7	450.4	78.6	573.8	62.0	313.7	86.5	1070.0	50.2	482.2
	80 - 84	1365.2	1026.2	1146.3	561.4	524.3	1212.8	379.0	565.5	266.9	769.8	228.7	424.3	518.1	1399.6	442.2	792.2	70.4	584.8	88.0	471.4	60.5	996.9	47.5	544.0
	≥ 85	1817.0	1707.7	2678.5	1230.8	548.3	1608.6	670.9	972.5	370.8	1238.3	565.8	947.7	736.6	2809.2	1184.8	2018.7	129.2	1188.4	211.4	1035.5	91.3	1729.1	109.2	1110.3
	**Total**	13407.5	423.3	7004.1	185.9	5157.1	445.7	2124.6	154.6	2379.1	321.0	1359.3	155.8	4810.7	540.5	2746.6	253.0	810.6	340.3	541.4	200.3	903.5	643.2	433.1	263.4
	30 - 34	117.2	43.8	0.0	0.0	12.4	12.7	0.0	0.0	22.4	40.7	0.0	0.0	46.7	55.5	0.0	0.0	16.3	88.7	0.0	0.0	13.2	104.1	0.0	0.0
	35 - 39	205.1	69.8	58.6	19.1	136.6	128.6	11.4	10.2	30.8	49.1	10.3	15.8	42.8	47.9	21.4	22.5	7.5	35.4	7.5	37.0	6.0	41.4	6.0	40.1
	40 - 44	452.8	133.3	62.1	17.0	144.9	119.0	20.7	15.7	74.7	100.1	0.0	0.0	146.0	142.2	38.9	34.6	61.3	251.4	0.0	0.0	27.4	167.4	5.5	31.1
	45 - 49	839.9	228.6	128.0	32.0	419.5	311.1	37.3	25.4	117.7	147.9	0.0	0.0	298.1	273.5	78.9	64.9	30.7	117.7	12.3	46.2	34.6	193.3	4.9	26.1
	50 - 54	1262.9	370.2	256.9	68.4	540.5	417.6	124.7	87.2	277.5	359.9	15.0	18.0	422.3	448.7	93.8	88.3	54.7	222.6	16.4	65.2	48.5	304.7	17.6	100.3
	55 - 59	1496.0	450.2	270.3	71.8	703.2	543.0	87.9	60.2	244.4	315.8	46.2	53.8	461.6	541.3	124.0	123.0	77.1	312.7	24.1	91.5	85.4	553.3	3.9	22.5
2021	60 - 64	1655.7	519.6	392.1	107.8	634.7	516.9	165.0	119.1	332.0	428.1	57.2	66.4	531.3	681.1	149.2	155.9	125.3	506.0	29.2	111.0	90.8	585.0	13.5	79.2
	65 - 69	1534.9	527.3	508.5	150.0	533.4	489.1	220.9	175.1	379.0	519.0	121.5	149.2	516.9	722.3	131.7	145.1	113.4	506.8	35.5	144.7	60.0	398.5	22.8	141.9
	70 - 74	1448.6	545.4	682.2	216.5	486.8	510.9	178.7	160.5	330.3	494.8	145.0	188.6	583.9	843.6	310.8	350.6	79.3	390.5	38.2	161.7	49.7	354.2	35.5	244.8
	75 - 79	1317.8	656.3	852.9	326.6	394.7	579.2	218.9	247.3	359.2	687.0	190.9	285.1	432.0	788.3	344.2	468.0	108.6	694.5	77.9	373.5	62.8	632.8	41.8	372.2
	80 - 84	1210.7	849.0	1086.8	532.5	394.4	839.0	294.4	434.7	308.1	785.5	250.5	453.1	402.3	1083.6	412.7	762.2	107.8	870.5	109.7	601.0	44.2	644.2	47.1	542.0
	≥ 85	1773.3	1481.8	2778.6	1200.5	528.2	1360.3	639.3	857.9	421.6	1250.3	665.4	1019.7	658.4	2215.1	1293.1	2152.9	157.2	1342.5	235.8	1092.8	70.0	1229.4	67.8	675.2
	**Total**	13314.9	405.8	7077.0	185.9	4929.5	410.7	1999.2	144.3	2897.8	376.7	1502.1	169.8	4542.1	501.9	2998.9	275.9	939.4	381.2	586.6	213.9	592.5	370.5	266.5	151.0
