# Supplementary material for: Mortality burden of cardiovascular disease attributable to ambient PM2.5 exposure in Portugal, 2011 to 2021
Source: BMC Public Health. 2024 Apr 27;24:1188. doi: 10.1186/s12889-024-18572-0 (PMC11055300; doi:10.1186/s12889-024-18572-0)
Supplement: Supplementary file 3 — Supplementary Material 3. [file 12889_2024_18572_MOESM3_ESM.pdf]

Table S3: Years of life lost (YLL) and years of life lost rate per 100K (YLL rate) related to environmental burden of disease, by different years (2011-2021), age-groups, and sexes for Portugal and its different regions for stroke.

Year	Age group	Portugal				North				Centre				Lisbon Metropolitan Area				Alentejo				Algarve			
		Males		Females		Males		Females		Males		Females		Males		Females		Males		Females		Males		Females	
		YLL (EBD)	YLL rate (EBD)	YLL (EBD)	YLL rate (EBD)	YLL (EBD)	YLL rate (EBD)	YLL (EBD)	YLL rate (EBD)	YLL (EBD)	YLL rate (EBD)	YLL (EBD)	YLL rate (EBD)	YLL (EBD)	YLL rate (EBD)	YLL (EBD)	YLL rate (EBD)	YLL (EBD)	YLL rate (EBD)	YLL (EBD)	YLL rate (EBD)	YLL (EBD)	YLL rate (EBD)	YLL (EBD)	YLL rate (EBD)
2011	30 - 34	194.2	56.2	105.9	29.2	87.7	69.3	52.6	39.5	50.9	67.3	17.0	21.5	38.4	37.6	19.2	17.5	0.0	0.0	13.4	53.9	15.9	99.3	0.0	0.0
	35 - 39	226.5	59.0	145.6	36.0	64.3	45.2	80.4	53.4	46.7	56.1	15.6	17.7	88.0	77.9	35.2	29.2	24.5	89.2	12.3	44.8	0.0	0.0	0.0	0.0
	40 - 44	485.3	134.3	147.1	38.7	116.9	84.4	58.5	39.6	84.9	104.1	56.6	66.5	176.1	177.3	32.0	30.6	55.7	214.0	0.0	0.0	39.8	250.3	0.0	0.0
	45 - 49	662.5	185.5	357.8	93.6	263.3	187.2	131.7	87.0	140.2	170.3	38.2	43.7	187.5	202.9	144.2	143.4	50.2	188.8	20.1	75.2	12.0	78.5	23.9	149.4
	50 - 54	957.4	283.1	461.0	126.9	317.1	242.5	140.9	100.8	216.0	270.2	147.8	175.1	308.7	357.2	167.2	171.9	62.7	239.0	9.0	34.1	42.7	287.1	0.0	0.0
	55 - 59	1530.6	498.4	603.9	177.8	569.1	496.4	238.0	188.1	300.4	410.8	160.2	205.1	476.0	581.2	147.3	152.8	94.6	396.3	31.5	129.3	75.2	554.2	18.8	131.7
	60 - 64	1795.4	626.4	1055.6	326.9	555.8	546.6	286.9	253.4	442.5	665.2	312.4	422.1	520.4	621.9	402.6	407.9	170.8	781.6	27.3	115.5	65.1	505.0	32.6	244.0
	65 - 69	2473.7	1032.7	1600.6	553.9	768.6	964.5	547.9	566.9	589.2	1033.1	390.4	559.2	775.2	1055.1	425.1	485.4	179.8	961.6	139.2	608.5	124.4	1167.6	62.2	516.6
	70 - 74	3777.3	1839.2	2983.8	1145.1	1274.3	1920.3	1034.6	1207.3	976.9	1832.8	744.9	1101.0	974.3	1689.1	794.6	1099.9	346.2	1886.0	240.4	1004.5	114.6	1182.4	108.8	986.7
	75 - 79	4718.0	2707.4	4860.8	1983.2	1723.2	3090.9	1677.6	2111.6	1265.6	2650.1	1280.3	1941.5	1088.1	2442.2	1332.3	2066.3	367.0	2028.5	339.9	1391.3	165.7	2043.9	151.9	1407.2
	80 - 84	4863.9	4348.6	6391.8	3516.0	1518.2	4303.6	2094.9	3601.7	1477.0	4655.8	1803.6	3601.4	1168.8	4257.3	1744.6	3644.4	373.7	3138.8	448.5	2517.7	210.2	3830.1	192.4	2446.1
	≥ 85	5264.3	7153.9	11282.3	7205.7	1844.4	8395.2	3718.3	7737.9	1444.1	6764.7	3200.6	7164.4	1258.1	7013.7	3145.3	7539.5	390.3	4696.5	674.6	4548.9	212.4	5283.2	389.8	5338.6
	**Total**	26949.2	846.3	29996.2	813.2	9103.1	788.8	10062.3	756.4	7034.6	933.5	8167.5	934.3	7034.6	802.3	8167.5	805.5	2115.4	837.8	1956.1	692.2	1077.9	746.5	980.4	611.7
2012	30 - 34	44.9	13.6	104.8	30.1	14.5	12.0	29.1	22.6	0.0	0.0	47.6	63.0	16.3	17.0	0.0	0.0	0.0	0.0	0.0	0.0	11.4	75.1	22.8	141.8
	35 - 39	260.6	68.5	109.7	27.1	53.3	38.2	40.0	26.9	87.3	105.9	14.5	16.6	74.7	66.2	59.8	49.2	42.6	154.0	0.0	0.0	0.0	0.0	0.0	0.0
	40 - 44	324.2	89.4	236.9	61.8	109.0	79.0	72.6	49.0	26.4	32.5	39.7	46.3	149.4	147.6	95.1	88.6	29.1	110.9	19.4	76.1	9.5	59.8	9.5	56.4
	45 - 49	494.3	139.7	292.1	76.8	163.6	117.9	98.2	65.2	131.0	161.4	47.6	55.1	134.6	145.2	97.9	97.0	26.2	100.8	17.5	66.3	34.3	225.7	25.7	158.4
	50 - 54	1032.2	303.3	501.1	136.4	408.7	308.5	184.9	129.7	255.0	317.2	95.6	111.8	272.9	316.9	141.9	146.1	62.3	235.1	54.5	204.9	30.6	205.5	15.3	98.2
	55 - 59	1253.6	405.0	512.0	149.9	360.0	308.5	180.0	140.4	337.0	456.5	121.7	154.0	452.0	555.6	144.3	151.7	68.6	286.1	41.1	165.6	47.1	343.7	20.2	140.4
	60 - 64	1621.7	554.9	719.1	218.2	594.2	564.7	215.4	182.9	356.9	519.4	178.4	235.9	416.7	502.9	258.3	261.3	160.4	715.5	59.4	251.5	64.2	492.7	11.7	86.0
	65 - 69	2175.3	891.5	1259.7	428.0	800.7	980.4	504.4	511.1	557.6	969.5	275.4	393.1	601.3	803.9	339.6	376.9	141.2	737.6	100.9	436.6	69.3	637.7	29.7	239.7
	70 - 74	2885.1	1413.0	2153.1	833.2	914.6	1384.2	747.4	883.3	924.5	1768.6	604.9	904.6	738.9	1261.1	598.1	819.4	234.1	1337.6	150.5	653.4	69.8	716.0	53.4	489.3
	75 - 79	4004.5	2294.8	3861.8	1576.6	1314.3	2336.5	1293.3	1616.2	1201.3	2535.9	1137.1	1738.8	1083.6	2399.9	1060.1	1643.3	302.3	1707.5	309.0	1275.9	102.3	1274.7	66.0	610.8
	80 - 84	4529.0	3885.4	5556.6	2956.5	1615.4	4384.0	1872.1	3106.4	1327.3	4054.5	1543.8	3004.1	1072.2	3703.6	1480.6	2972.3	335.4	2722.8	470.6	2563.7	150.6	2636.3	158.3	1949.2
	≥ 85	4842.3	6403.7	10068.6	6235.3	1665.4	7339.0	3186.0	6409.6	1542.6	7075.6	3248.6	7147.3	1026.5	5522.8	2691.4	6157.7	392.4	4629.0	700.4	4632.8	171.5	4223.4	267.8	3575.5
	**Total**	23467.8	737.3	25375.5	685.3	8013.7	693.5	8423.4	629.7	6746.9	897.2	7355.0	840.7	6039.2	686.7	6967.1	665.6	1794.6	712.8	1923.3	682.0	760.6	526.8	680.4	422.1
2013	30 - 34	44.0	13.9	29.3	8.7	27.9	23.8	0.0	0.0	0.0	0.0	30.1	41.3	0.0	0.0	0.0	0.0	0.0	0.0	0.0	0.0	12.2	83.4	0.0	0.0
	35 - 39	120.9	32.6	134.3	33.7	63.9	47.2	38.4	26.4	13.8	17.2	55.2	64.1	30.0	27.2	15.0	12.5	0.0	0.0	12.7	46.4	11.2	63.5	11.2	60.3
	40 - 44	329.6	91.1	256.3	66.1	104.6	76.5	23.2	15.6	87.9	109.0	75.3	87.7	150.2	146.7	95.6	86.6	0.0	0.0	34.7	135.0	0.0	0.0	30.5	176.6
	45 - 49	549.9	157.2	384.9	101.8	178.0	130.5	167.6	112.1	101.8	127.0	67.8	79.1	196.8	213.0	110.7	109.7	20.8	81.2	31.3	120.7	54.9	361.2	9.1	56.6
	50 - 54	853.5	250.4	392.4	105.9	308.3	230.7	140.1	96.7	211.8	263.9	60.5	70.1	230.4	267.8	164.6	169.8	92.9	352.2	27.9	104.9	16.3	112.3	8.2	52.1
	55 - 59	1019.8	326.2	440.7	127.4	345.6	291.4	131.7	100.6	231.0	308.4	124.4	155.2	290.0	358.9	145.0	152.7	114.6	469.0	24.6	97.0	43.1	309.7	21.6	147.4
	60 - 64	1587.5	547.1	741.3	226.2	549.0	520.5	263.8	223.5	415.7	604.0	238.7	315.6	418.8	521.1	192.6	199.1	177.4	785.9	35.5	149.9	37.4	289.1	18.7	137.5
	65 - 69	1919.7	762.5	1150.5	379.6	599.2	699.2	393.4	381.8	418.3	706.9	241.8	337.8	625.7	822.7	383.9	414.8	222.8	1138.2	84.3	362.9	74.0	656.9	58.2	457.3
	70 - 74	2618.8	1282.0	1807.3	698.6	852.9	1290.6	612.1	725.1	617.6	1204.1	482.2	730.2	807.4	1342.6	489.2	654.4	264.6	1554.8	204.7	909.5	100.8	1035.0	35.1	318.3
	75 - 79	3645.0	2068.4	3534.9	1444.3	1152.9	2019.3	1140.8	1416.5	1122.9	2358.4	988.0	1521.3	947.0	2054.8	994.4	1533.8	349.0	2015.1	357.0	1503.4	105.6	1303.3	91.6	856.2
	80 - 84	3771.6	3130.0	4712.1	2439.3	1329.2	3486.8	1628.7	2637.0	1158.9	3450.2	1327.4	2514.0	912.6	3020.2	1271.8	2478.2	310.5	2467.7	388.1	2050.6	87.2	1456.0	136.3	1628.7
	≥ 85	4478.1	5720.1	8798.0	5241.2	1528.1	6451.2	3079.7	5939.9	1314.4	5862.5	2511.8	5357.7	1117.4	5783.2	2328.8	5083.1	412.3	4721.0	712.2	4565.1	133.7	3240.1	238.6	3091.6
	**Total**	20938.2	659.6	22382.2	603.0	7039.7	609.8	7619.5	567.3	5694.1	760.3	6203.2	708.9	5726.3	653.5	6191.6	589.6	1964.9	783.1	1912.8	679.8	676.4	469.6	658.8	407.1
2014	30 - 34	13.2	4.3	53.0	16.3	9.5	8.4	19.1	15.8	0.0	0.0	13.6	19.3	0.0	0.0	0.0	0.0	0.0	0.0	13.4	61.3	0.0	0.0	0.0	0.0
	35 - 39	182.0	50.8	48.5	12.5	52.5	40.4	0.0	0.0	25.0	31.9	12.5	14.8	89.8	83.7	25.7	21.8	0.0	0.0	0.0	0.0	0.0	0.0	10.6	59.0
	40 - 44	253.7	69.8	176.5	44.8	55.7	41.0	39.8	26.6	56.7	70.9	45.4	52.5	105.0	99.8	58.3	51.0	22.4	86.3	11.2	42.8	0.0	0.0	9.7	54.6
	45 - 49	407.4	118.2	238.5	63.7	86.0	64.7	35.8	24.3	102.2	129.4	61.3	72.6	115.6	125.1	94.6	93.8	70.6	279.7	40.3	158.6	8.7	57.3	0.0	0.0
	50 - 54	753.5	219.9	398.9	106.2	185.3	137.6	121.4	82.1	218.8	272.7	45.6	52.2	206.2	237.2	121.9	124.4	54.0	205.6	45.0	169.2	31.1	213.6	23.3	147.0
	55 - 59	1038.5	330.3	523.2	149.8	230.8	192.6	157.6	118.7	240.9	320.0	72.3	89.1	338.5	418.8	165.1	173.5	118.8	485.7	47.5	187.0	41.1	294.1	27.4	186.0
	60 - 64	1217.9	419.7	649.5	197.0	263.4	247.0	156.1	129.5	306.1	441.6	139.2	183.6	357.7	454.2	257.6	268.6	96.1	424.5	34.3	144.0	106.8	832.7	17.8	129.3
	65 - 69	1757.5	681.1	964.9	314.3	364.4	408.1	256.7	243.6	454.8	750.5	259.9	360.3	570.9	743.5	242.9	259.2	198.1	999.7	99.1	430.2	65.5	566.7	25.2	197.1
	70 - 74	2313.9	1121.4	1637.8	626.7	562.9	839.2	398.2	465.4	607.1	1199.7	435.7	665.2	639.4	1025.6	448.1	582.4	212.5	1278.5	173.9	786.2	112.7	1162.4	58.5	520.5
	75 - 79	3251.4	1833.1	2868.9	1166.6	786.0	1368.9	659.1	812.6	841.8	1775.8	719.8	1109.0	958.6	2022.7	837.3	1270.8	310.4	1830.8	380.3	1627.2	114.1	1390.9	77.2	725.8
	80 - 84	3188.8	2558.0	4044.4	2041.6	802.6	2022.8	999.0	1576.0	886.0	2538.5	1129.6	2083.0	748.3	2411.5	1086.4	2081.1	378.5	2937.6	396.5	2030.3	116.8	1895.3	124.6	1423.9
	≥ 85	3807.2	4714.1	7817.7	4499.0	926.2	3766.5	1820.2	3366.5	1114.3	4854.4	2334.3	4865.1	900.2	4486.3	2202.0	4621.2	396.6	4421.0	693.6	4294.8	172.4	4126.0	225.3	2846.8
	**Total**	18184.9	574.1	19421.8	521.9	4325.3	375.6	4663.0	345.6	4853.6	650.8	5269.1	602.6	5030.3	572.8	5539.9	524.9	1858.0	749.0	1935.0	691.2	769.2	534.8	599.6	368.7
	30 - 34	43.2	14.7	43.2	13.9	26.0	23.8	0.0	0.0	0.0	0.0	13.5	20.0	15.9	18.7	31.9	34.3	0.0	0.0	0.0	0.0	0.0	0.0	0.0	0.0
	35 - 39	92.3	26.7	26.4	7.0	11.9	9.5	11.9	8.8	12.4	16.2	0.0	0.0	43.8	42.6	0.0	0.0	22.6	90.0	11.3	44.0	0.0	0.0	0.0	0.0
	40 - 44	431.5	117.7	179.8	45.0	151.8	112.0	54.2	36.1	45.0	56.0	67.4	77.3	159.4	147.9	39.8	33.8	20.5	78.6	10.3	38.9	48.4	285.3	0.0	0.0
	45 - 49	529.2	154.2	270.0	72.2	205.2	156.2	87.9	60.2	81.0	103.2	91.1	108.1	143.6	154.0	59.8	58.6	74.0	295.5	9.2	36.6	0.0	0.0	10.9	66.1
	50 - 54	655.1	191.5	423.9	112.2	174.3	129.4	130.7	87.4	153.5	191.8	99.3	113.6	202.8	232.7	128.1	130.0	49.5	192.4	16.5	62.8	58.3	403.7	38.9	244.5

	55 - 59	1069.3	336.0	364.9	103.4	299.4	246.2	107.5	79.9	190.9	250.6	79.6	96.8	404.3	495.0	122.2	127.6	87.2	352.1	36.3	142.0	68.5	489.1	8.6	57.1
2015	60 - 64	1345.7	461.9	588.3	176.5	432.4	400.1	192.9	156.8	248.2	356.2	124.1	161.3	431.8	555.7	179.2	188.7	138.5	605.6	50.4	209.1	51.9	398.9	22.3	157.3
	65 - 69	1791.4	682.1	1079.9	349.3	604.2	658.8	304.9	285.5	362.8	588.5	292.6	407.5	560.2	722.3	331.9	350.2	117.5	588.3	64.1	280.0	94.4	805.6	56.7	441.0
	70 - 74	2250.8	1064.2	1836.9	682.3	660.0	950.3	589.8	660.3	504.5	985.4	451.1	678.8	716.6	1108.3	504.5	628.4	181.6	1099.6	177.2	814.6	125.3	1295.0	41.8	366.9
	75 - 79	3309.1	1874.4	3072.1	1265.2	1000.4	1747.4	981.6	1228.7	802.9	1727.8	701.5	1100.6	944.2	1961.9	856.7	1299.1	281.1	1712.3	316.7	1391.7	167.7	2025.5	96.4	920.4
	80 - 84	3603.9	2814.9	4334.3	2148.7	1132.4	2755.9	1435.2	2213.1	874.9	2447.6	1028.7	1874.4	973.2	3047.2	1244.2	2343.0	333.3	2547.2	355.3	1793.1	152.6	2471.7	113.6	1252.4
	≥ 85	4210.5	4972.0	8719.6	4808.1	1265.4	4884.1	2607.6	4609.0	1067.7	4440.3	2194.5	4388.7	1121.8	5313.9	2674.1	5369.7	417.8	4541.5	658.9	3920.0	173.9	3933.6	316.0	3869.3
	**Total**	19331.9	610.9	20939.2	561.6	5963.7	518.0	6504.4	480.9	4343.7	583.2	5143.6	588.3	5717.5	650.4	6172.4	582.1	1723.4	701.0	1706.1	614.5	941.0	655.2	705.0	430.7
	30 - 34	26.2	9.3	52.4	17.6	13.2	12.5	26.4	23.9	0.0	0.0	0.0	0.0	0.0	0.0	29.5	33.0	10.2	50.3	0.0	0.0	0.0	0.0	0.0	0.0
	35 - 39	132.1	39.9	96.1	26.6	36.3	30.3	24.2	18.4	62.8	85.5	31.4	39.9	27.0	27.4	27.0	24.7	0.0	0.0	9.4	38.5	0.0	0.0	0.0	0.0
	40 - 44	283.9	77.2	207.5	51.5	120.9	89.8	44.0	29.5	19.0	23.7	76.1	86.9	61.4	56.2	73.7	61.0	51.0	194.2	0.0	0.0	22.6	130.6	11.3	60.7
	45 - 49	393.5	114.2	285.3	75.7	118.8	90.7	108.9	74.6	145.8	186.0	42.9	50.6	66.4	69.7	88.5	84.8	7.7	30.7	23.0	91.7	40.8	268.1	20.4	121.6
	50 - 54	623.1	182.7	237.0	62.7	212.0	159.0	97.2	65.1	175.9	222.0	38.2	44.1	138.1	155.9	78.9	78.9	27.4	107.7	6.8	26.1	54.6	375.9	18.2	114.2
	55 - 59	912.2	283.1	456.1	127.0	319.0	256.8	124.5	90.2	202.1	262.5	94.3	112.6	304.2	372.3	182.5	190.1	54.2	215.8	30.1	116.4	24.0	170.3	24.0	157.5
2016	60 - 64	1192.4	409.4	643.1	192.3	411.2	378.7	209.0	168.0	198.5	283.0	116.8	151.0	429.3	557.9	256.1	271.1	104.4	460.1	36.5	151.8	41.7	323.8	27.8	195.9
	65 - 69	1597.9	593.9	926.9	294.3	509.3	533.7	297.6	269.8	376.6	598.5	193.3	266.4	530.7	680.5	326.1	340.3	101.9	496.0	84.2	364.7	58.9	484.9	11.8	89.5
	70 - 74	2088.2	965.0	1640.4	594.9	744.8	1034.5	597.7	649.6	476.5	920.3	287.6	429.8	593.6	894.0	577.7	691.6	150.6	903.1	102.8	473.6	83.1	869.9	73.3	633.8
	75 - 79	2859.3	1645.7	2616.9	1097.3	975.7	1738.7	910.9	1163.2	627.1	1388.1	617.2	990.3	843.1	1725.9	723.9	1096.8	233.1	1520.6	197.7	913.0	125.6	1519.6	109.9	1076.8
	80 - 84	3335.9	2557.6	3854.7	1877.7	1121.1	2669.7	1191.9	1793.9	855.8	2386.8	901.8	1625.0	886.6	2665.7	1170.0	2161.0	230.7	1743.2	328.9	1639.2	161.0	2644.5	179.3	1959.2
	≥ 85	3917.5	4380.5	7981.1	4233.4	1338.0	4798.1	2727.1	4625.9	946.6	3735.6	1893.1	3636.9	1037.1	4704.0	2395.1	4642.4	303.3	3181.9	536.0	3073.0	197.2	4262.9	291.2	3432.1
	**Total**	17362.2	549.4	18997.4	509.0	5920.2	514.7	6359.3	469.6	4086.8	550.8	4292.8	491.9	4917.5	558.2	5929.0	556.4	1274.4	522.1	1355.4	491.3	809.6	565.7	767.2	467.7
	30 - 34	14.2	5.2	56.7	19.9	12.8	12.6	38.5	36.2	0.0	0.0	12.5	20.9	0.0	0.0	0.0	0.0	0.0	0.0	0.0	0.0	0.0	0.0	0.0	0.0
	35 - 39	181.9	57.0	116.9	33.6	82.2	71.0	58.7	46.1	22.8	33.1	11.4	15.6	43.3	46.0	43.3	41.0	11.7	51.0	0.0	0.0	13.5	91.2	0.0	0.0
	40 - 44	224.4	61.0	224.4	55.7	74.8	56.2	96.1	65.3	20.8	26.3	41.5	48.3	91.9	83.3	78.8	64.4	10.6	40.1	0.0	0.0	24.6	140.4	0.0	0.0
	45 - 49	489.4	140.7	244.7	64.1	105.8	80.8	57.7	39.3	149.7	191.2	65.5	76.6	189.3	193.2	82.8	77.0	9.6	38.2	19.2	76.7	22.2	145.5	11.1	65.7
	50 - 54	569.5	168.2	351.2	93.0	188.8	143.4	128.8	86.6	116.8	151.1	58.4	68.6	147.7	165.1	105.5	104.5	34.2	138.3	25.7	99.8	59.4	408.6	19.8	122.6
	55 - 59	1045.1	322.0	409.7	112.7	340.3	270.4	158.8	112.8	227.9	294.9	73.5	85.9	269.6	329.1	111.6	115.7	90.5	357.1	45.2	173.3	69.7	494.0	0.0	0.0
2017	60 - 64	1296.8	441.1	601.3	178.5	380.0	343.7	144.1	114.1	312.1	435.8	140.1	177.1	394.7	511.9	225.5	240.2	104.5	459.9	39.2	160.5	52.9	407.9	37.8	265.5
	65 - 69	1426.7	519.9	1027.0	319.2	483.9	490.6	328.1	285.9	265.0	409.2	254.1	344.4	417.1	535.1	294.0	304.3	122.0	582.7	55.5	241.2	83.4	683.6	51.3	384.5
	70 - 74	2130.8	962.1	1534.4	545.2	608.6	820.9	534.8	567.9	488.6	911.0	309.3	451.9	691.5	1012.7	430.8	498.4	174.7	1020.2	142.5	650.6	90.4	927.1	47.8	402.0
	75 - 79	2817.8	1624.0	2514.7	1060.3	807.5	1438.8	792.7	1022.3	695.0	1601.8	543.8	895.9	842.5	1678.9	733.2	1094.4	247.5	1694.4	214.2	1023.9	106.8	1291.2	128.1	1276.2
	80 - 84	3344.5	2546.0	3778.8	1831.0	931.8	2192.7	1146.8	1702.4	847.4	2372.3	886.4	1611.0	898.8	2638.7	1135.0	2086.9	334.5	2552.3	334.5	1665.9	178.5	2993.2	115.7	1262.0
	≥ 85	4442.4	4755.3	8607.3	4390.2	1356.5	4639.9	2540.0	4118.7	1024.4	3720.8	2164.5	3865.8	1181.0	5103.3	2537.6	4718.4	466.0	4744.6	718.4	3948.4	207.0	4341.9	269.4	3092.4
	**Total**	17983.4	569.4	19467.1	520.7	5373.0	467.0	6025.1	443.2	4170.6	566.8	4561.2	525.4	5167.5	584.8	5778.1	539.5	1605.8	661.7	1594.3	581.7	908.3	638.5	680.9	415.5
	30 - 34	65.5	24.7	26.2	9.4	36.9	37.1	0.0	0.0	23.4	40.0	11.7	19.6	0.0	0.0	14.8	17.8	0.0	0.0	0.0	0.0	0.0	0.0	0.0	0.0
	35 - 39	108.0	34.8	36.0	10.6	56.4	49.8	0.0	0.0	32.2	46.7	10.7	14.6	13.6	14.8	0.0	0.0	0.0	0.0	7.5	33.6	0.0	0.0	13.6	86.2
	40 - 44	109.1	30.2	174.6	43.8	20.5	15.7	61.5	42.5	29.2	37.1	39.0	45.3	37.1	34.1	24.7	20.3	0.0	0.0	13.6	50.6	24.7	142.2	24.7	132.4
	45 - 49	393.2	112.3	226.1	58.4	157.0	119.9	64.7	43.7	52.7	67.3	26.3	30.8	122.5	122.2	77.9	70.0	30.6	121.9	24.5	96.7	11.1	71.4	22.2	128.6
	50 - 54	683.9	203.2	342.0	90.9	197.8	152.0	107.1	72.3	125.4	162.1	54.8	64.4	168.8	187.3	178.7	175.9	70.9	290.8	0.0	0.0	79.3	541.3	9.9	61.6
	55 - 59	973.2	298.5	401.6	109.3	348.4	273.8	145.2	101.2	207.1	267.9	103.5	121.0	297.4	360.7	113.7	117.4	33.6	133.5	9.6	36.8	61.1	440.5	17.5	112.2
2018	60 - 64	1365.3	458.5	602.3	176.2	364.8	324.1	182.4	141.4	311.0	434.2	119.6	151.2	515.4	667.0	181.9	192.4	66.6	287.8	45.8	184.4	75.6	573.5	45.4	314.8
	65 - 69	1647.5	603.2	937.4	292.3	549.9	555.9	277.6	240.8	299.5	462.5	198.0	268.3	534.0	698.7	347.4	364.4	91.9	437.9	42.4	185.0	122.0	1012.8	51.4	385.4
	70 - 74	1930.8	843.1	1276.2	439.4	553.2	710.8	349.6	354.7	420.8	784.6	315.6	461.1	682.7	976.2	458.7	514.0	128.9	737.0	55.7	252.7	69.2	683.5	63.9	525.0
	75 - 79	2455.6	1411.7	2440.4	1026.2	814.3	1445.6	792.9	1023.0	561.2	1293.4	615.3	1013.8	677.1	1303.3	728.5	1054.7	160.0	1133.1	120.0	587.2	119.7	1468.4	81.3	804.6
	80 - 84	2955.0	2219.1	3329.9	1611.0	861.5	1985.5	1081.7	1588.8	774.6	2168.5	811.3	1474.4	799.4	2266.8	918.8	1674.4	202.2	1577.0	213.1	1084.1	158.9	2662.5	132.4	1460.6
	≥ 85	4046.6	4141.2	8000.3	3923.0	1264.9	4090.6	2481.9	3857.2	936.1	3399.9	1955.0	3491.7	1196.4	4953.3	2535.5	4569.3	272.2	2686.5	426.1	2232.7	179.9	3614.6	279.8	3101.5
	**Total**	16733.7	530.4	17793.0	474.9	5225.6	453.8	5544.7	406.0	3773.1	512.8	4261.0	490.9	5044.4	570.0	5580.8	518.2	1056.8	439.4	958.2	351.5	901.5	634.7	741.9	453.7
	30 - 34	59.7	22.8	23.9	8.8	31.9	32.3	10.6	10.4	11.4	19.5	11.4	19.6	13.3	17.8	0.0	0.0	0.0	0.0	0.0	0.0	0.0	0.0	0.0	0.0
	35 - 39	65.7	21.5	98.5	29.8	9.7	8.7	9.7	8.0	0.0	0.0	31.4	43.9	48.9	54.5	24.4	24.2	0.0	0.0	12.9	59.3	13.3	95.1	13.3	86.8
	40 - 44	99.5	28.1	69.7	17.9	35.4	27.8	35.4	25.1	9.5	12.2	28.5	33.8	33.3	31.4	0.0	0.0	5.9	22.8	0.0	0.0	12.1	71.8	0.0	0.0
	45 - 49	376.5	105.9	215.2	54.4	87.8	66.6	39.9	26.7	51.4	65.3	94.2	109.1	190.2	183.3	60.1	51.7	21.1	83.6	5.3	20.4	21.8	136.0	10.9	61.3
	50 - 54	607.7	181.7	247.9	66.3	227.7	178.0	78.3	53.4	145.2	188.4	38.2	45.4	160.7	177.5	107.1	105.0	14.1	58.1	14.1	56.4	38.8	264.5	0.0	0.0
	55 - 59	690.3	209.3	366.3	98.0	219.4	169.8	106.6	72.6	161.6	208.1	107.7	124.1	228.1	272.1	110.1	111.9	24.9	98.3	8.3	31.7	34.2	245.6	25.6	162.6
2019	60 - 64	1184.1	393.7	470.0	135.7	331.3	289.8	119.5	91.1	268.3	370.8	70.0	87.2	354.4	456.4	156.7	164.4	68.3	293.7	43.1	172.6	118.5	898.7	59.3	407.6
	65 - 69	1512.9	552.5	777.2	240.0	447.2	446.1	253.6	214.9	396.1	606.6	158.4	213.7	399.1	530.1	266.1	279.9	97.6	463.8	21.3	92.1	88.0	736.6	62.9	466.6
	70 - 74	1894.1	804.5	1155.4	391.8	493.0	606.5	355.4	351.8	435.1	790.6	246.3	356.5	661.7	932.7	369.2	407.0	126.4	712.1	68.3	312.7	93.8	901.8	62.6	511.0
	75 - 79	2160.2	1224.8	1935.9	803.7	571.1	993.7	546.6	694.0	527.6	1229.5	511.1	847.7	693.4	1282.8	620.2	868.9	140.1	1013.4	109.7	544.8	129.8	1604.8	54.4	528.7
	80 - 84	2705.7	2015.5	2943.4	1415.3	720.2	1647.3	801.0	1167.0	742.8	2091.3	806.6	1465.8	837.9	2297.3	948.3	1693.0	160.4	1284.1	136.8	707.3	116.7	1930.5	142.6	1591.9
	≥ 85	3654.2	3567.5	7256.1	3437.4	1000.8	3066.8	2023.2	3019.8	966.9	3357.3	1955.1	3379.3	1100.4	4349.3	2295.2	4035.2	220.7	2087.6	397.4	1997.4	190.8	3722.2	249.5	2637.2
	**Total**	15010.7	474.4	15559.3	413.8	4175.6	361.1	4379.8	319.2	3716.0	503.4	4059.0	467.6	4721.4	531.6	4957.5	457.3	879.4	366.6	817.2	300.1	857.8	606.4	681.1	415.7
	30 - 34	65.4	24.9	10.9	4.0	13.2	13.4	0.0	0.0	29.4	48.8	9.8	16.5	24.2	32.6	0.0	0.0	0.0	0.0	0.0	0.0	0.0	0.0	0.0	0.0
	35 - 39	59.9	20.2	89.9	28.2	24.2	22.2	12.1	10.3	9.0	13.5	36.0	51.6	22.2	25.6	33.3	34.5	6.8	32.5	6.8	33.0	0.0	0.0	0.0	0.0
	40 - 44	136.2	39.5	118.0	31.1	43.9	35.5	0.0	0.0	40.9	52.8	40.9	49.1	40.4	39.5	40.4	34.8	6.2	24.7	18.5	72.1	8.3	51.3	8.3	46.8
	45 - 49	359.9	99.7	114.5	28.5	128.6	97.3	9.9	6.6	58.9	74.0	36.8	41.9	154.5	144.4	45.4	37.9	22.3	87.0	11.1	42.4	15.0	90.5	7.5	41.2
	50 - 54	445.0	133.1	299.1	79.9	203.0	160.0	105.9	72.9	91.9	119.8	59.1	70.1	113.5	123.6	121.6	117.7	34.8	143.4	5.0	19.8	20.1	136.6	26.8	163.4
	55 - 59	739.1	224.2	385.6	102.4	310.9	240.8	101.1	68.1	167.8	216.3	115.7	132.6	214.2	254.3	150.0	151.3	48.1	193.9	17.5	67.6	29.5	213.8	11.8	74.8

2020	60 - 64	1063.6	349.0	389.8	111.2	343.5	296.1	168.4	126.5	285.7	389.6	60.2	73.7	371.3	472.3	123.8	129.0	72.0	306.0	26.5	105.5	20.4	154.9	30.7	206.9
	65 - 69	1370.8	498.9	865.0	264.2	463.1	456.2	371.6	308.5	297.9	453.1	153.2	202.8	457.0	616.0	283.7	301.2	109.4	514.6	67.6	288.3	78.1	647.9	30.4	220.0
	70 - 74	1755.4	733.4	1230.4	414.2	649.3	778.6	521.4	509.4	395.1	706.1	225.8	327.6	618.3	862.2	435.4	474.3	80.0	448.4	72.0	331.9	97.1	931.0	46.8	380.6
	75 - 79	2194.3	1213.2	1961.3	791.3	784.4	1318.1	841.6	1023.4	507.3	1167.9	430.8	705.0	762.7	1358.7	594.7	800.3	120.0	876.5	117.9	596.2	109.8	1358.3	72.2	693.5
	80 - 84	2353.9	1769.5	2975.3	1457.3	913.7	2113.5	1214.4	1812.0	598.9	1727.4	691.0	1281.6	671.6	1814.0	850.3	1523.2	146.0	1212.4	229.0	1226.1	105.1	1732.9	94.0	1075.5
	≥ 85	3466.2	3257.6	6853.4	3149.2	1303.4	3823.8	2593.5	3759.6	912.1	3046.0	1660.3	2781.1	956.3	3647.5	2172.2	3701.0	246.7	2267.9	453.2	2220.4	140.1	2654.1	224.5	2282.5
	**Total**	14009.6	442.4	15293.2	405.9	5181.3	447.8	5939.7	432.2	3394.8	458.0	3519.4	403.3	4406.2	495.0	4850.7	446.7	892.4	374.6	1025.2	379.2	623.7	444.0	553.0	336.3
2021	30 - 34	33.1	12.4	66.2	24.7	0.0	0.0	12.8	13.0	11.6	21.0	11.6	21.6	24.2	28.7	24.2	27.7	0.0	0.0	16.9	104.1	0.0	0.0	0.0	0.0
	35 - 39	60.7	20.6	101.1	32.9	0.0	0.0	23.6	21.0	21.3	33.9	21.3	32.8	22.2	24.8	44.3	46.5	7.8	36.7	7.8	38.3	6.3	42.9	6.3	41.6
	40 - 44	229.8	67.6	101.1	27.6	53.5	44.0	32.1	24.3	48.3	64.7	58.0	73.5	120.8	117.7	10.1	8.9	7.1	28.9	0.0	0.0	11.4	69.4	5.7	32.3
	45 - 49	364.4	99.2	173.9	43.5	115.7	85.8	67.5	46.0	95.7	120.3	52.2	60.7	154.2	141.5	45.4	37.3	25.4	97.6	12.7	47.9	0.0	0.0	5.1	27.1
	50 - 54	502.3	147.2	199.4	53.1	189.3	146.2	77.4	54.1	108.7	140.9	38.8	46.6	202.3	214.9	89.0	83.7	17.0	69.2	11.3	45.0	18.3	114.9	0.0	0.0
	55 - 59	702.7	211.5	344.9	91.6	325.9	251.6	144.0	98.6	177.8	229.7	75.2	87.6	199.6	234.1	135.5	134.3	25.0	101.3	10.0	37.9	24.1	156.4	8.0	46.6
	60 - 64	919.0	288.4	439.8	120.9	354.6	288.8	151.0	109.0	231.1	298.0	83.0	96.3	278.0	356.4	160.6	167.8	60.6	244.7	43.3	164.4	38.4	247.1	17.4	102.7
	65 - 69	1277.9	439.0	641.3	189.2	473.8	434.4	262.0	207.7	332.0	454.5	145.9	179.1	409.0	571.5	220.2	242.5	91.8	410.2	44.1	179.9	38.5	255.7	11.8	73.6
	70 - 74	1495.7	563.1	1019.6	323.7	508.3	533.3	332.7	298.8	404.4	605.8	279.3	363.2	543.3	785.1	373.8	421.6	112.7	554.5	63.9	270.6	19.6	139.9	27.0	186.1
	75 - 79	1746.7	869.9	1724.4	660.4	620.0	909.7	601.4	679.5	452.2	864.7	462.3	690.2	565.7	1032.4	534.3	726.4	124.8	797.8	154.1	738.9	65.1	656.1	49.3	438.4
	80 - 84	1899.8	1332.2	2316.7	1135.1	686.8	1460.9	890.8	1315.2	573.0	1460.7	606.7	1097.4	521.7	1405.3	689.3	1273.0	147.7	1192.5	189.4	1037.9	59.5	868.2	61.0	702.4
	≥ 85	3029.2	2531.3	5846.1	2525.9	1066.8	2747.6	1964.6	2636.3	847.2	2512.7	1659.2	2542.7	834.3	2807.0	1819.5	3029.3	304.4	2599.1	483.0	2238.3	92.1	1618.6	179.7	1790.3
	**Total**	12261.2	373.7	12974.6	340.7	4394.6	366.1	4559.9	329.1	3303.2	429.4	3493.3	394.9	3875.4	428.2	4146.2	381.4	924.2	375.0	1036.6	377.9	373.2	233.4	371.4	210.4
